# Why do people buy dogs with potential welfare problems related to extreme conformation and inherited disease? A representative study of Danish owners of four small dog breeds

**DOI:** 10.1371/journal.pone.0172091

**Published:** 2017-02-24

**Authors:** P. Sandøe, S. V. Kondrup, P. C. Bennett, B. Forkman, I Meyer, H. F. Proschowsky, J. A. Serpell, T. B. Lund

**Affiliations:** 1 University of Copenhagen, Department of Food and Resource Economics, Frederiksberg C., Copenhagen, Denmark; 2 University of Copenhagen, Department of Large Animal Sciences, Frederiksberg C., Copenhagen, Denmark; 3 La Trobe University, Department of Psychology and Counseling, Bendigo, VIC, Australia; 4 Danish Kennel Club, Solrød Strand, Denmark; 5 University of Pennsylvania, School of Veterinary Medicine, Department of Clinical Studies, Philadelphia, United States of America; University of Missouri Columbia, UNITED STATES

## Abstract

A number of dog breeds suffer from welfare problems due to extreme phenotypes and high levels of inherited diseases but the popularity of such breeds is not declining. Using a survey of owners of two popular breeds with extreme physical features (French Bulldog and Chihuahua), one with a high load of inherited diseases not directly related to conformation (Cavalier King Charles Spaniel), and one representing the same size range but without extreme conformation and with the same level of disease as the overall dog population (Cairn Terrier), we investigated this seeming paradox. We examined planning and motivational factors behind acquisition of the dogs, and whether levels of experienced health and behavior problems were associated with the quality of the owner-dog relationship and the intention to re-procure a dog of the same breed. Owners of each of the four breeds (750/breed) were randomly drawn from a nationwide Danish dog registry and invited to participate. Of these, 911 responded, giving a final sample of 846. There were clear differences between owners of the four breeds with respect to degree of planning prior to purchase, with owners of Chihuahuas exhibiting less. Motivations behind choice of dog were also different. Health and other breed attributes were more important to owners of Cairn Terriers, whereas the dog’s personality was reported to be more important for owners of French Bulldogs and Cavalier King Charles Spaniels but less important for Chihuahua owners. Higher levels of health and behavior problems were positively associated with a closer owner-dog relationship for owners of Cavalier King Charles Spaniels and Chihuahuas but, for owners of French Bulldogs, high levels of problems were negatively associated with an intention to procure the same breed again. In light of these findings, it appears less paradoxical that people continue to buy dogs with welfare problems.

## Introduction

The selective breeding of dogs has created highly specialized breeds for hunting, herding and guarding but also breeds with extreme physical features like a very short nose, a flat skull, very small body size, protruding eyes, a highly sloping croup and the like. Many of these breed-specific traits were cultivated and refined using inbreeding which, together with a lack of selection pressure against health issues in some breeds, has resulted in high levels of inherited diseases. Thus, dog breeding–whether due to anatomic features or genetic disease load–has led to a number of health and other welfare problems for many purebred dogs [1–6]. These breeding-related welfare problems among purebred dogs have been a matter of concern among professionals for decades, and some research has been conducted on the issue, along with initiatives instigated by breed clubs, veterinarians, animal welfare organizations, and politicians to raise awareness of the problems [7]. In light of this, one might also expect a reaction from the market in terms of prospective dog owners not being willing to buy purebred dogs from breeds known to be seriously affected by these kinds of problems.

However, the demand for purebred dogs with extreme physical features and with high loads of inherited diseases does not in general seem to be decreasing. On the contrary, some of these dog breeds appear to be increasing in popularity among dog owners in the western world. Examples include the English Bulldog, French Bulldog, Pug, Cavalier King Charles Spaniel, and Chihuahua [8–10]. In 2015, the French bulldog was the sixth most popular dog breed in America [9], and the third most popular dog breed in the United Kingdom [11]. Similarly, the number of yearly registrations of Chihuahuas in the Danish Dog Registry doubled in five years, from 1,487 in 2007 to 3,132 in 2011, so that by the end of the period it was the third most popular breed in Denmark (H. F. Proschowsky, personal communication).

Different explanations have been proposed regarding the apparent paradox, that people buy breeds of dog that are predisposed to diseases and other welfare problems, while at the same time caring deeply about their dogs. One line of thought is that prospective dog owners, prior to acquisition, are not fully aware of the potential problems their dog may face. It is also possible that dog owners simply do not perceive the clinical signs of some inherited disorders as problems, but rather as normal, breed-specific characteristics [12]. Alternatively, it could be that, when choosing a suitable breed, other characteristics of the dog may be considered more important than its health and welfare [13]. Dogs with extreme physical features may possess qualities that matter to their owners to such an extent that they outshine any health and other welfare problems faced by the dogs.

Previous research has examined several possible explanations for why dog owners choose specific dogs, although the amount of variance explained is often small and the results inconclusive. For example, at a societal and cultural level, it has been suggested that specific dog breeds flourish as part of fashion and that the media serve to amplify these developments. Herzog [14] examined shifts in preferences for some types of dogs, and suggested that social contagion is a major factor determining dog breed preferences. An example of this is the celebrity-driven fad for extra-small varieties of dogs such as Chihuahuas [15–16].

People may also use particular dog breeds to express aspects of their own personality and preferences [17–18]. Studies have found resemblance in facial features between owners and their dogs [19], and resemblance in overall physical features between owner and dog, e.g. size, hair, attractiveness, perceived friendliness [20–21]. Infantile facial features and other human-like attributes in dogs have also been found to attract people [22–23]. Finally, a dog’s physical appearance may influence social acknowledgment from other people [24].

The specific relationship between owner and dog could further explain why certain animals are more likely to become objects of human attraction [25]. In this context, Archer and Monton [22] discovered a positive correlation between owner attachment to their dogs or cats and preferences for images of animals with infant features (large forehead, large and low-lying eyes, and bulging cheeks). Hoffman and others [26] and Serpell [27] also found that some behavioral characteristics are related to levels of owner attachment to their dogs, although Ghirlanda and others [28] found no evidence that breed-related differences in behavior influenced the popularity of different breeds.

An array of previous studies thus indicates that both physical and behavioral attributes of dogs may have an impact on how attractive a specific breed or breed characteristic is perceived to be. However, as far as we are aware, no previous study has investigated the motivational patterns behind peoples’ choices between dog breeds, or how these relate to the quality of the relationship between owners and dogs of specific breeds. To address this issue, we surveyed a representative sample of owners of four different breeds of dogs (two with extreme phenotypes, one with a high load of inherited diseases and one relatively healthy) with the overall goal of examining their motivations for acquiring their dog, the health and behavior problems encountered, and the quality of relationships between the dog owners and their dogs.

Our study was conducted in Denmark, with dog-owning participants being recruited through the Danish Dog Registry (DDR); membership of which is a legal requirement for all privately owned dogs. Each dog is required to be micro-chipped and registered by the age of eight weeks, and identified by breed and the owner’s address. During the period from 2009 to 2014, approximately 70000 new puppies entered the DDR annually. Of these, one third were registered as purebred by the Danish Kennel Club (DKC), close to one fifth were registered as mixed breed, and the rest were registered as either a breed without a known pedigree (the majority) or with a pedigree from another parallel organization issuing its own pedigrees.

Our study aimed to answer the following questions: 1) Do motivations for acquiring a dog, and pre-purchase owner characteristics, differ between owners of the four breeds? 2) Do levels of expenditure on veterinary treatments and health and behavior problems experienced differ for owners of the four dog breeds? 3) Do motivations prior to acquisition, and owners’ experiences of health and behavior problems with their dogs, explain differences in the quality of the owner-dog relationship between the four breeds? 4) Do intentions of acquiring the same breed the next time a dog is to be procured change as a function of experienced health and behavior problems?

## Method

### Choice of breeds

We selected three small dog breeds that can be considered extreme in different ways, and one control breed. The breeds were specifically chosen among family dog breeds and not breeds used for hunting, guarding, herding etc. We use the term ‘small’ to denote family dogs that, in size, are at the smaller end of the scale. The breeds were selected using a combination of scientific literature and “breed profiles” provided by the Swedish insurance company Agria (S1–S8 Files). The proportion of Swedish dogs with life and/or health care insurance is very high (76.5%) [29] and the quality of the data has been validated by Egenvall and others [30]. The calculation of rates and relative risks in the breed profiles are based on “dog years at risk” (DYAR) and each individual breed is compared to “all breeds”, which for the latest version of the breed profiles, represents 1.35 million DYAR. Sweden and Denmark are very closely related and the prevalence of diseases in the dog populations of the two neighboring countries is considered equal. Table 1 summarizes characteristics concerning morbidity and mortality from the breed profiles.

**Table 1:** Mortality and morbidity characteristics from Agria breed profiles used to select the four study breeds.

Breed	Relative Risk of death*	Median age (years) at death ♂/♀	Relative Risk of at least one VCE**	Median age (Years) at first VCE ♂/♀
All breeds	1	6.6/7.0	1	4.9/5.6
Cairn Terrier	1	9.1/9.9	1	7.7/7.9
Cavalier King Charles Spaniel	1.3	7.8/7.9	1.2	4.6/5.4
Chihuahua	0.8	3.9/4.2	1	2.8/2.9
French Bulldog	1.2	2.5/3.8	1.9	1.6/1.8

* Relative risk compared to all breeds. Mortality rates include events where, most commonly, a veterinarian assigned the cause of death and some cases (generally acute or accidental death) where the owner and a witness confirmed the death of the dog.

** Relative risk of at least one Veterinary Care Event (VCE) compared to all breeds. A VCE represents a visit to the veterinarian where the cost exceeds the self-risk.

The Chihuahua and the French Bulldog were selected to represent breeds that have experienced an increase in popularity over a short period of time and which suffer from health problems related to their conformation. The Chihuahua is the smallest breed in the world and can be bred down to 500g, even though the ideal weight is between 1.5 and 3 kg [31]. There are several health and welfare problems in this breed related to its small size including patella luxation [32–33], dystocia [34–35], and high levels of aggression associated with fear [36–37]. In addition, the breed exhibits a high prevalence of fractures due to its delicate and fragile conformation. The relative risk of death due to injury is up to 28 times the risk of all breeds (S2 File). The median age of death and the median age at first veterinary care event is low compared to all breeds (Table 1). The fact that the overall relative risk of death is lower than for all breeds might reflect that the population of insured Chihuahuas is generally young due to the dramatic increase in popularity (Brenda Bonnett, personal communication).

The French Bulldog belongs to the so-called brachycephalic breeds, characterized by a flattened facial profile. The anatomic malformations of the nasal cavity and upper airways lead to impaired breathing, which can develop into Brachycephalic Obstructive Airway Syndrome [4]. The relative risk of death due to a range of respiratory problems is 14–70 times the risk of all breeds according to the Agria insurance data (S6 File). Protruding eyes is another potentially problematic breed characteristic and the insurance data reveals a relative risk of veterinary care events because of corneal trauma or corneal ulcers of 10–18 times the risk of all breeds (S7 File). Besides being flat faced, French bulldogs are born with screwed bobtails due to a malformation of the vertebrae in the tail (hemivertebrae). If this malformation expands to the spine, it can cause pain and neurological signs due to spinal cord compression [38]. The breed also has a high prevalence of dystocia [39]. French bulldogs are very young when they exhibit their first veterinary care event (less than two years compared to around five years for all breeds in common, Table 1). This might reflect that the health problems of the breed are related to congenital malformations instead of acquired and age related diseases.

The Cavalier King Charles Spaniel was selected to represent a breed with a relatively stable popularity and a distinctive cute look, even though the breed suffers from a high number of life-threatening inherited disorders, as pointed out by numerous research papers as well as television programs such as ‘Pedigree Dogs Exposed’, aired by the British television channel BBC1 in August 2008. The breed has been maintained based on very few founder individuals and, probably due to inbreeding, inherited disorders such as syringomyelia, affecting the brain and spinal chord [40], the heart disease Myxomatous Mitral Valve Disease (MMVD) [41], and hearing problems due to Primary Secretory Otitis Media (PSOM) [42], are widespread in all populations around the world. Heart disease is the number one cause of death in the breed, with a relative risk of dying from heart failure more than ten times the average for all breeds (S3 File). Long-term medical treatment of MMVD has become possible and this is probably a contributory cause of the high median age at death of the Cavalier King Charles Spaniel (Table 1).

The Cairn Terrier was selected to represent a breed in the same size range and functional category (companion animal) but without extreme conformation and with the same level of disease load as the overall dog population based on Agria breed profiles (S4 and S5 Files). The Cairn Terrier is not free from inherited disorders and diseases, with glaucoma [43] and craniomandibular osteopathy [44] having been described in this breed. However, the relative risk of death and VCE (Veterinary Care Event) equals “all breeds” (RR = 1) and the high median age at death and median age at first veterinary care event reflect that the breed is generally healthy (Table 1).

### Sample and recruitment

The Danish dog market is characterized by the absence of large commercial breeding operations (e.g. puppy farms) found in other countries. The majority of the 70,000 puppies that are purchased each year come from smaller breeders with 2–4 breeding dogs. This is partly due to tradition and partly to legislation. If a Danish breeder produces more than 2 litters per year, s/he will be subject to the Commercial Dog Breeding Act, meaning that s/he must fulfill specific requirements regarding registration, inspection, education, etc. [45]. Also, Danish pet shops are not allowed to sell puppies. In addition to puppies bred in Denmark, there is some importation—legal as well as illegal—of puppies from Eastern Europe. This is particularly the case with fashionable breeds such as the Chihuahua and French Bulldog. Under current law, imported dogs are also listed in the DDR.

More than half of the Cairn Terriers that enter the DDR have a DKC pedigree. The percentage for Cavalier King Charles Spaniels and French Bulldogs is 30% and 20% respectively, while less than 10% of Chihuahuas have a DKC pedigree (H. F. Proschowsky, personal communication).

We requisitioned a complete list from the DDR with names and addresses of all persons who had registered one of the four selected dog breeds in the period April 2009 to October 2014. This October 2014 threshold was selected so that the dog would be at least 6 months old at the time of data collection, implying that the dog would be integrated in family life and that possible welfare problems would have been more likely to appear and be observed by the owner. For each breed, a random sample of 750 persons was drawn, resulting in a total sample of N = 3000. An invitation to participate in the survey was sent May 18, 2015 to the 3000 selected persons. The questionnaire could either be answered electronically or by mail. On June 13 a reminder was sent to persons who had not yet responded electronically to the questionnaire or requested a postal questionnaire. The survey was closed for web and postal participation on July 27, 2015.

Permission to receive and use the data was applied for with the board of the DDR and it was granted conditional on permission from the Danish Data Protection Agency. Such permission was applied for, and the Data Protection Agency replied in writing that permission was not required for this kind of study but that we still had to comply with the Danish Data Protection Law. Additionally, we sought ethical and legal advice from a member of the Faculty of Law of the University of Copenhagen on how best to ensure compliance with Danish legislation. On this basis the administrator of the DDR gave us access to names and addresses of relevant dog owners. Prospective participants were contacted by means of a posted letter and, for those who did not respond in the first round, by a reminder letter. In both letters, it was made clear that all participants would remain anonymous and that all information delivered would be treated confidentially. Subsequently all replies were anonymized and the file with names and addresses of Danish dog owners deleted. It was clear that participation was voluntary; and the participants were seen to give their implicit consent by deciding to participate either online or by means of returning a filled out printed questionnaire by post. This is the normal way of proceeding in Denmark for surveys of this kind. At the time when we conducted our study, ethics committees covered only biomedical research and the University of Copenhagen did not have Institutional Review Boards.

### Survey design and measures

The invitation letter specified to the respondent that the study aimed to cast light on the owner-dog relationship and to gain knowledge as to why and how people choose a dog, so that future dog owners could get relevant guidance in their choice of dogs. In the questionnaire, respondents were asked about socio-demographic details, whether he/she had had a pet earlier in life, descriptive details about the dog (e.g. breed, current age, purchase price), possible welfare problems, the choice of dog and breed, their expectations of a good dog, daily life with and care of the dog, expenses of the dog, owner-dog attachment, whether there were other dogs in the household and about intended future procurement of a dog. An overview of the entire questionnaire is provided in Table 2. Respondents were instructed to have the specific dog in mind they had registered with the DDR during the last five years when responding to the dog-related questions. If they had more than one dog of the same breed, which were registered in the same period, they were instructed to have the oldest of these dogs in mind. This was done to make sure that possible respondents that procured a dog of the same breed after October 2014 would not think of that dog.

**Table 2:** Overview of themes covered in the questionnaire.

Overall theme	Details
Socio-demographic	• Gender • Postal code • Education • Household characteristics • Self-reported population-density of living area • Housing • Education • Employment status • Income • Professional work with dogs
Pet career	• Pets in childhood
About the dog	• Breed • Female/male • Neuter status • Still has dog • Where is the dog today? • Why do you not have it anymore? • Main responsibility for dog • Dog’s age when procured • Current age of dog • Purchase price • Pedigree
Welfare problems and veterinary assistance	• Welfare problems not requiring veterinary assistance • Welfare problems requiring veterinary assistance • Veterinary visits during last year (and for what)
Choice and procurement of dog	• Who in household got the idea to procure the dog? • Other influential persons or media in choice of dog • Who suggested the dog breed? • Influential persons or media in choice of breed • Who has most contact with dog in daily life? • Where was the dog procured? • Planning of procurement • Factors affecting choice of dog
Expectations of a good dog	• Easy to train • Easy to handle • Good around people/children/dogs • Appropriate levels of activity • Ensures that owner gets exercise
Daily life with and care of dog	• Hours that the dog is alone on weekdays • Professional dog walker • How do you take the dog outside? • How often do you or others take the dog for a walk? • Activities with the dog • How often do you or others train the dog? • Dog brought along on vacation • Dog brought along at work • Dog brought along at social visits
Expenses of dog	• Type of insurance • Expenses on veterinary care • Expenses on dog food • Expenses on toys, snacks etc.
LAPS questions	• Degree of attachment to dog
Other dog currently, earlier dogs, dogs in the future, other pets currently	• Other pets in household • Number of dogs in household • Earlier number of dogs • A new dog after the current dog • The same breed as current breed? • Other pets in the household

We used a subset of the questionnaire items to develop relevant measures. For example, to create a measure aiming to assess whether choice of dog was carefully planned or more spontaneous, we identified respondents who indicated that “the choice of dog was incidental” in response to the question: “Who suggested the dog breed?”, and who indicated that there had “not really been any planning” in response to the question: “How much planning was there before the purchase of the dog?”. Two knowledge acquisition measures were developed: The first related to use of books/professionals and was a composite variable (range 0–4) based on whether relevant response options were ticked in two multiple response questions, the first being: “Which of the following things influenced your choice to purchase a dog?” (relevant response options were 1. “I/we read books about dogs”. 2. “I/we contacted professionals to learn more about the dog”). The second question was “Which of the following things influenced your choice of breed?” (where relevant response options were 3. “I/we read books about this breed” and 4. “I/we contacted professionals to learn more about different dog breeds”). The second knowledge acquisition measure related to whether friends/colleagues/family influenced the choice of breed, and whether the choice was influenced by the circumstance that the respondent had owned this dog breed before. These were response options offered to the question: “Which of the following things influenced your choice of breed?”. Further, we measured the point-of-purchase of the dog on the basis of the multiple response question “From where did you procure the dog?”. We report the following responses “From a breeder with several breeding dogs”, “from a breeder / family with only the bitch who is the mother of the dog”, “I/we got the dog from a previous owner”, and a final “other” category, where other responses that were infrequently reported were collapsed (“I raised the dog myself”, “other (non specified)”, “don’t know”, “from abroad”, and “from a shelter”). In an effort to identify motivational drivers for choice of dog, insofar as the characteristics of the dog are concerned, dog owners were asked to rate how important a number of attributes had been when choosing the dog (To what degree did the following factors influence the choice of dog? (Response options: 1 = not at all, 5 = very high degree). Examples of these attributes were: “facial expression”, “overall appearance”, “the health of the breed”, “it was easy to find a dog of this breed”.

Our measure of health and behavior problems, as experienced by the dog owners, included six problems requiring, and five problems not requiring, veterinary assistance, such as “Has your dog experienced any problems with vomiting and/or diarrhea, where it did not require a vet visit?”and “Has your dog been to the vet because of respiratory problems (e.g. coughing, wheezing or strange breathing sounds)?” Response categories were: “Never”, “One or a few times”, or “Many times”. A measure of annual veterinary expenditure was also employed; using a question in which respondents were asked how much money they usually spent on veterinary bills on a yearly basis (categories ranging from “0–999 Danish Kr.” (DKR) (0–150 USD) to “more than 20000 DKR” (more than 3.032 USD) and a “missing response”).

Due to its prior validation as a self-administered measure of attachment [46–47]), the Lexington Attachment to Pets Scale (LAPS) [48] was used in this study to quantify the emotional attachment participants felt towards their dog. Comprising items from the Pet Attitude Scale [49], the Companion Animal Bonding Scale [50] and the Pet Attitude Inventory [51] in addition to earlier scales, the LAPS represents a compendium scale that provides insight into a range of emotional, moral, and social aspects of owners’ attachments to their pets. The LAPS has been shown to have acceptable construct validity, good convergent validity with similar scales such as the Pet Attachment and Life Impact Scale [52] and high internal consistency, with a coefficient alpha reported between 0.92 and 0.99 [53].

In prior studies using the LAPS, respondents have been asked to indicate levels of agreement on a 4-point scale (0 = disagree strongly to 3 = agree strongly) to 23 statements (e.g. “My pet makes me feel happy”), with higher scores on the scale indicating higher owner attachment to their dog. In the present study, respondents were offered five response options because we included an intermediate “neither agree nor disagree” option so that undecided respondents would have a relevant response option. This modification did not damage the factorial validity of the LAPS, as Cronbach’s alpha for the overall scale was very acceptable (0.899). Acceptable alpha coefficient levels were also found for each of three LAPS subscales (General attachment 0.856, People substituting 0.849, Animal rights/welfare 0.706). Only the composite LAPS was used in the analyses we report, although the results were essentially similar when analyses (described below) were repeated for each of the three sub-scales.

Finally, respondents were asked whether they planned to procure a new dog at some future point and, if so, what breed they would select. We constructed five response options, “have no plan to”, “don’t know”, “yes, but not the same breed”, “yes, maybe the same breed”, and “yes, for sure the same breed”. Additional measures that were used primarily as control variables were the dog’s current age (7 response options from “0–1 year” to “more than 5 years”), the respondents’ age (collapsed into 10 gradient categories), gender, educational qualifications (5 categories from “compulsory school” to more than 4 years of higher education”), household composition (lives alone, two adults, family with child/children), type of accommodation (three options: in flat, in house with garden, farm/house in the countryside), and population density (five categories ranging from low to high density). Population density is based on the Eurostat standard for classification of geographical areas and the division in Denmark is based on a list of municipal codes [54].

### Data analysis

We calculated response rates for the total sample and for each dog breed, and then conducted a non-response analysis for each breed. In the non-response analysis, it was possible to compare the distribution of region, population density in the household area, and the age of the dog from the sample, with information from the DDR. The highest deviations observed are reported and, for each dog breed, it is reported whether there are statistically significant differences (at the 0.05 level) between the background population (census data from the DDR) and the sample. The NPAR (Chi^2^) test command in SPSS was used for this analysis.

To address the first research question (‘Do motivations for acquiring a dog, and pre-purchase owner characteristics, differ between owners of the four breeds?’), we examined differences in pre-purchase characteristics between owners of the four dog breeds. This was done through Generalized Linear Modeling (GLM) (with logit link in the case of binary dependent variables or poisson logit link in the case of count variables) where we also input the socio-demographic measures described earlier to control for confounding, along with the dogs’ ages to ensure that possible differences were not caused by recall bias attributable to differences in elapsed time since acquisition of the dog across the breeds. We report the results of Wald chi^2^ tests for differences between the dog owners. In order to simplify the analysis, motivational factors for choice of dog were subjected to a Principal Components Analysis (PCA). Factor loadings from direct oblimin rotation (where factors are allowed to correlate) of all components having eigenvalues greater than 1 are reported. For the subsequent analysis, the identified motivational factors were calculated as the sum of the raw scores of items that had factor loadings over 0.3, divided by the number of items (theoretical range for all motivation measures: 1–5). We chose to use raw scores instead of factor scores to increase the transferability of the study results, as raw scores are easily replicated in future studies [55]. To identify differences in motivational patterns between owners of the four different dog breeds, we ran GLMs (using normally distributed identity link) and also entered socio-demographic variables to control for possible confounding, and the dogs' age to take into account possible recall bias attributable to differences in elapsed time since acquisition of the dog across the breeds. We report Wald chi^2^ tests for differences between the dog owners.

To address the second research question (‘Do levels of expenditure on veterinary treatments and health and behavior problems experienced differ for owners of the four dog breeds?’), we recoded the items regarding health and behavior problems into dichotomies: 0 = “never/one or a few times”, 1 =“many times”. For each breed, prevalence rates are reported. Three composite measures were calculated. The first was the number of frequently occurring problems not requiring veterinary assistance, the second was the number of frequently occurring problems requiring veterinary assistance, and the third was the total number of frequently occurring problems requiring/not requiring veterinary assistance (see Table 7 for details). For all three composite measures, we tested whether the mean number of frequently experienced problems varied between breeds while controlling for dog age. This was done with GLM using poisson logit link, as the composite variables are count variables. It was also tested whether annual expenditure on veterinary assistance was different between the breeds while controlling for socio-demographic factors and dog age. This was done with GLM using a cumulative logit link, as the dependent variable was ordinal.

For testing the third research question (‘Do motivations prior to acquisition and owners’ experiences of health and behavior problems with their dogs explain differences in the quality of the owner-dog relationship between the four breeds?’) responses to the 23 LAPS items were summed using the scoring procedure 0 = disagree, 1 = partly disagree, 2 = neither agree nor disagree, 3 = partly agree 4 = agree, and after reversing the scores for two negatively worded items. This gives a possible scale range from 0 to 92. The sample range was 16–92, with a mean of 64.1 (sd. 15.8). To identify differences in attachment between owners of the four dog breeds, we ran GLMs (using normally distributed identity link) and also entered socio-demographic variables and dog age to control for possible confounding.

To examine whether the owner’s motivations for choosing their dog and their experiences of health and behavior problems with their dog explain the quality of the owner-dog relationship, GLMs (using normally distributed identity link) were conducted. In the model, motivational drivers behind choice of dog identified in PCA were entered as explanatory variables, as well as the number of frequently occurring disease or health problems requiring/not requiring vet assistance. Additional explanatory variables were inserted into the model to examine whether differences in the quality of the owner-dog relationship across breeds were explained by the degree of owner/family engagement with the dog. These included two dichotomous variables recording whether the respondent was mainly or jointly responsible for the dog and whether the respondent had the most contact with the dog (0 =“yes“, 1 =“no”). We also included a variable indicating the veterinary expenses related to the dog on an annual basis. The variable originally had five response options, but was recoded into three gradient levels. This recoding was undertaken only in the high expenditure end of the responses because only a small proportion of respondents used the response options: “5000–10000 DKR” (700–1500 USD), “10000–20000 DKR” (1500–3000 USD), and “Over 20000 DKR” (3000 USD or more)). The three gradient levels were (1 = “0–999 DKR” (0–149 USD), 2 = “1000–4999 DKR (150–699 USD), and 3 = “5000 DKR or more” (700 USD or more) and a further “missing” category, in order not to drop the 58 observations where there were missing responses to this question.

To address the fourth research question (‘Do intentions to acquire the same breed the next time a dog is to be procured change as a function of experienced health and behavior problems?’) we first compared differences between owners of the four dog breeds regarding their plans to acquire a new dog. This was done with a multinomial regression with the socio-demographic factors and dog’s age entered as control variables. Following that, we examined whether experienced health and behavior problems with the dog predicted the propensity to plan to acquire the same breed again (0 =“no for sure”; 1 = “yes for sure”). Logistic regression was used for this analysis. The main explanatory variable was the number of frequently occurring disease or health problems requiring/not requiring veterinary assistance (a measure described earlier). In addition, the LAPS score was employed as an explanatory variable. Socio-demographic factors and the age of the dog were also inserted to ensure that they were not confounding the association. This analysis was conducted separately for each breed. In any instance where health and behavior problems explained propensity to acquire the same dog again, the predicted probabilities were calculated (using Stata’s margins command where the other variables were set at their mean value) and displayed in graphs.

## Results

### Response rates and non-response analysis

Out of the 3000 recruitment letters distributed, no contact was made with 364 persons at the registered address, primarily because the addressee had moved, or due to an unknown address being supplied (registered by the Danish Postal service in a return letter). Of the remaining 2636 individuals, 911 responded to the questionnaire (796 web responses and 115 postal responses), giving an overall response rate of 35%. After removal of 24 owners who reported that they neither currently nor earlier had owned one of the four dogs, there were 883 respondents (see Table 3). The response rate varied across owners of the four dog breeds: Cairn Terriers, 45%; Cavalier King Charles Spaniels, 33%; Chihuahuas, 23%; and French Bulldogs, 31%.

**Table 3:** Overview of response rate and dog status.

	Cairn Terrier	Cavalier King Charles Spaniel	Chihuahua	French Bulldog
**Responses**				
Total invited	750	750	750	750
Reached contact ^A^	690	682	634	630
Completed questionnaire	309	228	148	198
Response rate	45%	33%	23%	31%
**Dog status**				
Still has dog	298 (96.4%)	220 (96.5%)	143 (96.6%)	185 (93.4%)
Dog is dead	8 (2.6%)	7 (3.1%)	3 (2.0%)	13 (6.6%)
Not reported	3 (1.0%)	1 (0.4%)	2 (1.4%)	0 (0%)

^A^ After subtraction of owners in which the postal address supplied from Danish Dog Register was invalid.

The overall response rate was good and, accordingly, non-response analysis showed a quite acceptable fit between available census data and the four dog breed samples. Only modest deviations from the background population were detected. The largest occurred in owners of Chihuahuas and French Bulldogs, in which there was a 4–5% over-representation of people from the Capital Region and 5–6% over-representation of people living in densely populated areas. Among owners of Chihuahuas, owners of older dogs (4–6 years) were over-represented by 5%. The opposite applied to owners of French bulldogs, where owners of dogs under 4 years were over-represented by approximately 4%. However, for all four parameters, and for owners of all four dog breeds, no statistically significant deviations between the background population and the sample were detected.

In the following analyses, owners whose dog had died (or where the status of the dog was unknown) are removed, giving a final sample size of 846 respondents.

### Pre-purchase characteristics and motivations for choice of dog breed

Owners of Chihuahuas were different from the other dog owners on several factors associated with pre-purchase characteristics (Table 4). A higher proportion of Chihuahua owners reported that there “wasn’t really any planning” before the acquisition, and that the “choice of dog was incidental”. Also, owners of Chihuahuas reported being significantly less inclined than owners of Cavalier King Charles Spaniels and French Bulldogs to acquire knowledge from books and professionals about dogs before the purchase decision.

**Table 4:** Pre-purchase characteristics and point of purchase (in percent or means)–per dog type.

	Cairn Terrier (N = 298)	Cavalier King Charles Spaniel (N = 220)	Chihuahua (N = 143)	French bulldog (N = 185)	Results of Chi^2^ Wald tests,p-value (chi^2^, df; N)*
**Planning**					
"Choice of dog breed was incidental"	5.4%	6.4%	11.9%	3.8%	< 0.05 (7.92; 3; 805)
"There wasn't really any planning"	14.4%	12.3%	28.0%	15.1%	< 0.01 (13.93; 3; 805)
**Knowledge acquisition**					
Mean (s.d.) From books/professional advice	0.33 (0.7)	0.57 (0.8)	0.24 (0.5)	0.48 (0.7)	< 0.001 (31.81; 3; 805)
“Friends/colleagues/family recommended this breed”	16.8%	20.0%	13.3%	11.9%	0.202 (4.62; 3; 805)
“I have had this dog breed before”	43,0%	20,9%	11,9%	13,0%	< 0.001 (58.87; 3; 805)
**Point of purchase** ^A^					
From a breeder with several breeding dogs	58.1%	52.3%	32.2%	34.6%	< 0.001 (39.83; 3; 815)
From a breeder / family with only the bitch who is the mother of the dog	32.2%	35.9%	32.9%	40.5%	0.377 (3.10; 3; 815)
I / we got the dog from a previous owner	6.0%	10.0%	21.7%	14.6%	< 0.001 (26.49; 3; 805)
Other (includes: raised it myself, from abroad, from shelter, other, don’t know)	4.4%	5.5%	14.7%	11.9%	< 0.001 (22.07; 3; 805)

* Wald chi^2^ tests from Generalized Linear Models (GLM) (using binomial logit link and poisson logit link in the case of ‘From books/professional advice’) as to whether there is significant difference between owners of the four dog breeds. Control variables included in the GLMs were gender and age of the respondent, age of the dog, education, household type, type of living residence, and population density.

^A^ This was a multiple response question, so percentages sum to more than 100.

Owners of Cairn Terriers appeared to be less inclined to acquire knowledge from books and professionals than owners of Cavalier King Charles Spaniels and French Bulldogs (results from poisson regression where Cairn Terriers were set to reference value). Conversely, owners of Cairn Terriers seemed to rely on prior experience with this breed to a much greater extent (45.9%) than was the case with owners of other breeds. Owners of Cairn terriers and Cavalier King Charles Spaniels were significantly more likely to have obtained their dog as a puppy directly from a breeder, while owners of Chihuahuas and, to some extent, French Bulldogs tended to acquire their dogs from a previous owner.

Principal components analysis extracted three motivational factors labeled, respectively, distinctive appearance, breed attributes, and convenience (Table 5). The questionnaire response, “the dogs’ personality” loaded on both the distinctive appearance and breed attributes factor, making its interpretation difficult. For this reason, we removed this item from the PCA, and report on it separately.

**Table 5:** Factors that are important when choosing a dog.

To what degree did the following factors affect the choice of your dog?	Distinctive appearance	Breed attributes	Convenience
The dog’s facial expression	.884		
The dog's overall appearance	.857		
The dog was different/unique	.608		
The color of the dog	.698		
The dog’s breed		-.750	
The characteristics / behavior of the breed		-.821	
The health of the breed		-.749	
It was easy to find a dog of this breed			.795
The dog was a bargain (it was a fair price)			.853

Results from principal component analyses (pattern matrix from direct oblimin rotation) (N = 821). Factor loadings below 0.300 are not reported.

There were differences in the motivational patterns behind choice of dog (Table 6). Owners of Chihuahuas were less motivated by the personality of the dog prior to acquisition, and breed attributes were less important to them compared to owners of the other breeds. Motivational factors related to convenience were more influential for owners of Chihuahuas compared to owners of the other dogs, although not at a significantly different level. In contrast, owners of Cairn Terriers were significantly more inclined to be motivated by breed attributes and less inclined to be motivated by the distinctive appearance of the dog. Owners of Cavalier King Charles Spaniels, but also of French Bulldogs, seemed to specifically value the dogs’ distinctive appearance and personality when acquiring these breeds.

**Table 6:** Motivational factors for owners’ choice of dog breed–per dog type.

	Cairn terrier (N = 290–297)		Cavalier King Charles spaniel (N = 214–218)		Chihuahua (N = 136–141)		French bulldog (N = 182)		Results of Chi^2^ Wald tests, p-value (chi^2^, df; N)*
	avg.	s.d.	avg.	s.d.	avg.	s.d.	avg.	s.d.	
Distinctive appearance of the dog	3.76	(1.27)	4.16	(1.39)	3.84	(1.62)	4.02	(1.46)	< 0.01 (12.35; 3; 787)
Breed attributes	4.10	(.82)	4.00	(.81)	3.41	(1.19)	3.88	(0.84)	< 0.001 (52.82; 3; 793)
Convenience	2.49	(1.13)	2.59	(1.09)	2.62	(1.29)	2.39	(1.06)	0.331 (3.43; 3; 793)
The dogs' personality	4.19	(0.99)	4.44	(0.98)	3.89	(1.36)	4.37	(1.02)	< 0.001 (19.62; 3; 808)

* Wald chi^2^ tests from GLMs (using normally distributed identity link) as to whether there is significant difference between owners of the four dog breeds. Control variables included in the GLMs were gender and age of the respondent, age of the dog, education, household type, type of living residence, and population density.

### Health and behavior problems

Owners’ experiences of different health problems with their dogs were divided between issues requiring veterinary care or other professional assistance and those where no professional treatment was needed (Table 7). Owners of French Bulldogs had the highest level of experiences with health and behavior problems with their dogs in both categories, while owners of Cairn Terriers had the lowest level of such experiences.

**Table 7:** Dog owners’ experiences with frequently occurring health and behavior problems.

	Cairn Terrier (N = 288–295)	Cavalier King Charles Spaniel (N = 212–219)	Chihuahua (N = 133–141)	French bulldog (N = 178–185)	Test Statistics
**Problems reported by owner to occur frequently, that did not require a visit to a veterinarian** ^A^					
Vomiting and/or diarrhea	11.2%	6.4%	7.8%	21.3%	Chi^2^ test < 0.001 (24.70; 3; 838)
Skin changes, ear problems, itching, problems with the anal glands	3.7%	12.5%	6.4%	14.8%	Chi^2^ test < 0.001 (22.18; 3; 833)
Coughing, wheezing or strange breathing sounds	4.4%	4.6%	16.3%	14.8%	Chi^2^ test < 0.001 (29.84; 3; 834)
Cramps, unsteadiness, problems with balance	0.3%	0.5%	2.2%	0.5%	Chi^2^ test = 0.178 (4.91; 3; 835)
Behavioral problems such as aggression, uncleanliness, fear of noises or of being alone	6.1%	4.6%	10.1%	5.5%	Chi^2^ test = 0.192 (4.74; 3; 831)
Mean (s.d.) number of frequently occurring problems not requiring veterinary assistance ^B^^,^ ^F^	0.25 (0.54)	0.29 (0.55)	0.42 (0.65)	0.53 (0.80)	Wald Chi^2^ test< 0.001 (29.10; 3; 819)
**Problems reported by owner to occur frequently that did require a visit to a veterinarian** ^C^					
Gastrointestinal problems (e.g. vomiting or diarrhea)	0.7%	2.3%	0%	1.6%	Chi^2^ test = 0.177 (4.93; 3; 838)
Skin problems (e.g. skin changes, itching, otitis, problems with the anal glands)	3.0%	9.2%	2.9%	9.2%	Chi^2^ test < 0.01 (14.51; 3; 838)
Respiratory problems (e.g. coughing, wheezing or strange breathing sounds)	0.7%	0.9%	1.4%	2.2%	Chi^2^ test = 0.499 (2.37; 3; 836)
Disease of the brain or in other parts of the nervous system (e.g. epilepsy, slipped disc)	0.3%	0.9%	0%	0%	Chi^2^ test = 0.378 (3.09; 3; 836)
Problems with the heart	0.3%	0.9%	0%	0%	Chi^2^ test = 0.382 (3.06; 3; 835)
Behavioral problems such as aggression, uncleanliness, fear of noises or of being alone	0%	0%	0%	0%	n.a.
Mean (s.d.) number of problems requiring veterinary assistance many times ^D^^,^ ^F^	0.05 (0.23)	0.14 (0.46)	0.04 (0.24)	0.13 (0.37)	Wald Chi^2^ test< 0.001 (16.58; 3; 829)
**Mean (s.d.) number of problems requiring/not requiring veterinary assistance** ^E^^,^ ^F^	0.30 (0.65)	0.43 (0.49)	0.47 (0.73)	0.66 (0.99)	Wald Chi^2^ test< 0.001 (30.74; 3; 811)

^A^ Proportion responding that the dog “many times” had problems with the disease or behavior in question

^B^ Composite scale of all problems not requiring assistance

^C^ Proportion responding that the dog has been to a veterinarian “many times” because of the disease in question

^D^ Composite scale of all problems requiring veterinary assistance

^E^ Composite scale of all problems reported.

^F^ Wald tests from poisson regression after adjusting for the dog’s age.

Owners of French Bulldogs were more inclined to report that their dog had experienced gastrointestinal and skin problems many times (that did not require veterinary treatment) compared to owners of the other three dog breeds. Owners of Cavalier King Charles Spaniels had the second highest level of experiences with skin problems with their dogs (that did not require veterinary treatment) and they were on a similar level to owners of French Bulldogs when veterinary treatment was necessary. Owners of Chihuahuas and French Bulldogs were more inclined to report that their dog had experienced respiratory problems many times (that did not require veterinary treatment) compared to owners of the other two breeds. Apart from gastrointestinal problems that did not require veterinary treatment, owners of Cairn Terriers had the lowest or second lowest rates of experience with the different health issues outlined in Table 7.

Owners of French Bulldogs reported the highest expenses for veterinary care and owners of Cairn Terriers the lowest after adjustment for dog age (Table 8). A high percentage (81.2%) of owners of Cavalier King Charles Spaniels had visited the veterinarian in order to obtain a health check for their dog within the last year, which represents the highest proportion among owners in this study. Owners of French Bulldogs showed the lowest proportion in this respect (67.0%), Cairn Terriers the second lowest (70.5%), and Chihuahuas the second highest (72.7%), although owners of French Bulldogs reported the highest number of incidents of sudden illness or injury (29.2%) compared to Cavalier King Charles Spaniels (18.8%), Chihuahuas (16.1%), and Cairn Terriers (13.4%). Owners of French Bulldogs also reported the highest number of incidents involving chronic/long term illness demanding veterinary treatment (8.6%) compared to Cavalier King Charles Spaniels (5.5%), Chihuahuas (4.2%), and Cairn Terriers (4.0%). Owners of Chihuahuas reported the highest level of treatment for dental problems including tooth cleaning (32.9%) compared to Cavalier King Charles Spaniels (16.5%), Cairn Terriers (11.1%), and French Bulldogs (4.3%).

**Table 8:** Expenditure on veterinary treatment during the last year–per dog type.

	Cairn Terrier (N = 281)	Cavalier King Charles Spaniel (N = 207)	Chihuahua (N = 130)	French bulldog (N = 174)	Results of Chi^2^ Wald tests p-value (chi^2^, df; N)*
**Annual veterinary expenses**^**A**^					
0–999 DKR (0–150 USD)	52.7%	36.2%	36.9%	34.5%	< 0.01 (13.39; 3; 805)
1000–4999 DKR (151–759 USD)	44.1%	57.5%	60.8%	53.4%	
5000 DKR or more (760 USD or more)	3.2%	6.3%	2.3%	12.1%	

* Wald chi^2^ tests from Generalized Linear Models (with cumulative logit link)) as to whether there is significant difference between owners of the four dog breeds. Control variables included in the GLM were gender and age of the respondent, age of the dog, education, household type, type of living residence, and population density.

^A^ Response to the question: “How much money do you usually spend on veterinary bills (if your dog is insured, please state the amount you spent, before the bills were paid by the insurance company)”

The level of health problems and the level of expenditure on veterinary treatment found in the four breeds conform to the authors’ expectations when choosing these breeds as subjects for study.

### Differences in the quality of the owner-dog relationship (LAPS)

Based on owners’ responses to the 23 LAPS items, there were statistically significant differences across breeds in the quality of the owner-dog relationship (Table 9). Owners of Cavalier King Charles Spaniels and French Bulldogs showed similar levels of attachment to their dogs. Owners of Chihuahuas experienced the highest level of attachment, whilst owners of Cairn terriers experienced the lowest level of attachment. When we investigated these breed differences further, we found many differences at the single question item level from the LAPS. For example, 61% of Chihuahua owners “strongly agreed” that “Dogs deserve just as much respect as humans do”, compared with 50% of French Bulldog owners, 47% of Cavalier King Charles Spaniel owners, and 35% of Cairn Terrier owners. Also, 70% of Chihuahua owners “strongly agreed” that “I would do almost anything to take care of my dog”. This compares with 62% of French Bulldog owners, 56% of Cavalier King Charles Spaniel owners, and 43% of Cairn Terrier owners.

**Table 9:** LAPS score–per dog type.

	Cairn terrier (N = 296)		Cavalier King Charles Spaniel (N = 214)		Chihuahua (N = 137)		French bulldog (N = 182)		Results of Chi^2^ Wald tests p-value (chi^2^, df; N)*
	avg.	(s.d.)	avg.	(s.d.)	avg.	(s.d.)	avg.	(s.d.)	
LAPS overall score	60.5	(16.0)	64.9	(16.2)	69.2	(14.3)	65.1	(14.8)	<0.01 (15.82; 3; 794)

* Wald chi^2^ tests from GLM (using normally distributed identity link) as to whether there is significant difference between owners of the four dog breeds. Control variables included in the GLM were gender and age of the respondent, age of the dog, education, household type, type of living residence, and population density.

Follow-up multivariate analysis showed that two of the motivational factors, distinctive appearance and breed attributes, were positively associated with the quality of the relationship for owners of the four dog breeds, i.e. owners that reported to be highly motivated by these factors were also found to be very attached to their dogs (Table 10). Higher rates of frequently occurring health and behavior problems experienced by owners were marginally positively associated with the quality of the relationship (p = 0.07). In addition, greater levels of engagement with the dog—i.e. being mainly responsible for the dog and having most contact with the dog—was associated with higher levels of owner-dog attachment. However, even after inclusion of these variables (along with socio-demographic control variables), Table 10 also reveals that this did not remove the significant differences in attachment between owners, as owners of Cavalier King Charles Spaniels and Chihuahuas have significantly higher attachment to their dogs compared with owners of Cairn terriers

**Table 10:** The influence of dog breed, extent of health/behavioral problems, motivational factors behind choice of dog, and engagement with dog on the quality of the owner-dog relationship (based on the LAPS where higher scores indicate higher quality)–results from GLM (dependent variable: LAPS) (N = 759)*.

	Coeff.	Std. Error	Wald chi^2^	Sig.
(Constant)	41.24	4.25	94.29	<0.001
**Dog breed (ref: Cairn terrier)**				
Cavalier King Charles Spaniel	3.67	1.33	7.66	<0.01
Chihuahua	4.82	1.71	7.95	<0.01
French Bulldogs	1.85	1.46	1.61	.205
**Number of frequently occurring health/behavioral problems**	0.93	0.66	2.01	.157
**Motivational factors behind choice of dog**				
Convenience	0.50	0.47	1.11	.293
Distinctive appearance	1.59	0.53	9.12	<0.01
Breed attributes	1.77	0.64	7.69	<0.01
**Engagement with the dog**				
Expenditure on veterinary assistance (ref: “0–999 DKR” (0–150 USD))				
1000–4999 DKR (151–759 USD)	1.75	1.09	2.55	.110
5000 DKR or more (760 USD or more)	4.47	2.37	3.56	.059
Expenditure not reported	-0.44	2.35	0.03	.853
Respondent not mainly responsible for the dog (ref: “no”)	-2.56	2.48	1.07	.301
Others in household have most contact with the dog (ref: “no”)	-5.56	1.99	7.81	<0.01

* Results are from GLM (using normally distributed identity link). Control variables included but not reported in the GLM were gender and age of the respondent, age of the dog, education, household type, type of living residence, and population density.

### Effects of health and behavior problems on acquisition of a new dog

Do experienced health and behavior problems affect an owner’s plan to acquire the same breed of dog next time? Owners of French Bulldogs had a clearly higher propensity to plan to acquire the same breed next time a dog was to be procured (29.2%) (Table 11) compared to owners of the three other dog breeds: Cavalier King Charles Spaniel (22.3%), Cairn Terrier (20.1%) and Chihuahua (17.5%). Owners of Chihuahuas (24.5%), meanwhile, were keener than owners of French Bulldogs (10.3%), Cairn Terriers (9.1%) and Cavalier King Charles Spaniels (8.6%) on the idea of acquiring another breed next time a dog was to be procured.

**Table 11:** Plan to acquire a new dog after the current dog.

	Cairn Terrier (N = 298)	Cavalier King Charles Spaniel (N = 220)	Chihuahua (N = 143)	French bulldog (N = 185)
Yes, for sure the same breed	20.1%	22.3%	17.5%	29.2%
Yes, maybe the same breed	19.5%	24.1%	21.0%	28.1%
Yes, but not the same breed (or not decided)	9.1%	8.6%	24.5%	10.3%
No, no plan to acquire new dog	21.1%	15.0%	14.0%	5.4%
Don’t know whether a new dog will be acquired	30.2%	30.0%	23.1%	27.0%

Test statistics from multinominal logit regression after adjustment for gender and age of the respondent, age of the dog, education, household type, type of living residence, and population density: Chi^2^ 29.37 (d.f. 12); p<0.01.

After collapsing propensity to acquire the same breed again into a binary response (1 = “yes for sure”; 0 = all other response options as reported in Table 11), we studied whether the extent of experienced health and behavior problems with the dog predicted propensity to respond “yes for sure” (Table 12). We report results regarding this in the form of coefficients from the logit regression, as the two main explanatory variables were continuous and we were interested in their possible gradient effects on plans to acquire the same breed again. Among owners of Cairn Terriers, Cavalier King Charles Spaniels and Chihuahuas, the number of experienced health and behavior problems (both those requiring and not requiring veterinary treatment) did not affect owners’ plans to acquire the same breed of dog. However, among owners of French Bulldogs the reported number of health and behavior problems did explain some of the propensity, as higher numbers of problems did decrease the propensity to plan to acquire the same breed next time.

**Table 12:** GLM results regarding what explains plans to acquire the same breed (“yes, for sure”)*.

	Cairn Terrier (N = 281)			Cavalier King Charles Spaniel (N = 204)			Chihuahua (N = 113)			French bulldog (N = 170)		
	Coeff.	Wald chi^2^	Sig.	Coeff.	Wald chi^2^	Sig.	Coeff.	Wald chi^2^	Sig.	Coeff.	Wald chi^2^	Sig.
(Intercept)	-27.08	0.00	1.00	-4.10	6.50	<0.05	-5.08	3.74	0.053	-6.50	11.17	<0.001
Number of frequently occurring health and behavior problems	-0.09	0.11	0.74	-0.11	0.24	0.63	-0.50	0.97	0.32	-0.59	5.85	0.02
LAPS score	0.04	12.17	<0.001	0.02	2.47	0.12	0.04	1.71	0.19	0.06	10.61	<0.01

* Wald chi^2^ tests from Generalized Linear Models (using logit link) whether there is significant difference between owners of the four dog breeds. Control variables included in the GLMs were gender and age of the respondent, age of the dog, education, household type, type of living residence, and population density.

**Fig 1:**
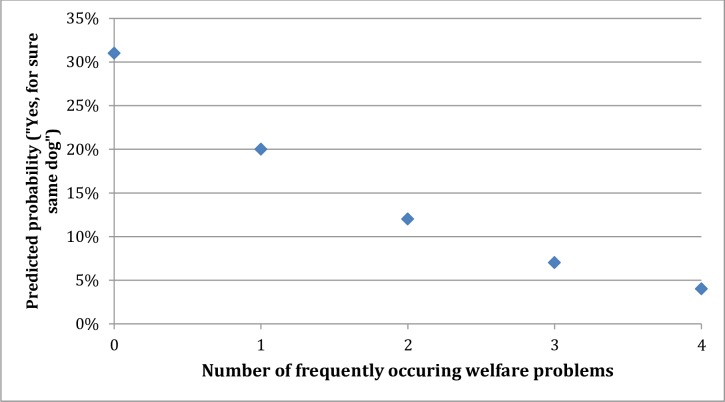
Predicted probability for acquisition of same dog breed. Predicted probability for responding “yes, for sure” regarding acquisition of same dog breed among owners of French Bulldogs.

Among owners of French Bulldogs, the probability of reporting “yes, for sure” dropped from 31% if there were no reported health and behavior problems to around 20% if there was one problem and to 12% if there were two problems (Fig 1). In practice, many French Bulldog owners did not report any problems; as seen in Table 7, the mean number of reported problems was 0.66 among owners of French Bulldogs. Overall, 62% of these owners did not report any problem, 15% reported one problem, 13.5% reported two problems, and the remaining 6.2% reported three or four problems. In other words, almost 80% of the owners of French Bulldogs reported one health or behavior problem at the most. At this level, the probability of reporting that they plan to acquire the same dog breed next time is 22% (cf. Fig 1).

## Discussion

The motivations that Danish people had prior to acquiring a dog, reported retrospectively, were found to differ across owners of the four breeds examined in this study (Chihuahuas, French Bulldogs, Cavalier King Charles Spaniels, and Cairn Terriers). While owners of one breed (Cairn Terriers) were mainly concerned about breed attributes, such as health, when acquiring a dog, owners of two of the other breeds did not seem to be much concerned about the health of the dog prior to acquisition. They were mainly interested in the dog’s distinctive appearance and personality (French Bulldogs) or seemed to prioritize that it was an easy dog to find and obtain (Chihuahuas). Owners of Cavalier King Charles Spaniels, in contrast, were motivated by the dog’s distinctive appearance when acquiring the dog, but also considered personality and other breed attributes, such as behavior and health. So, while potential owners of two of the studied breeds (Cairn Terriers and Cavalier King Charles Spaniels) may be interested in information about the health of the breed they plan to acquire, the potential owners of at least two other breeds appear to be less interested in this.

Also of interest, the proportion of owners of French Bulldogs and Cavalier King Charles Spaniels who planned to acquire the same breed again in the future was just as high, or higher, than the proportion of owners of Cairn Terriers. Furthermore, experiencing health and behavior problems with their dogs did not appear to have an impact on the owners’ likelihood of acquiring a dog of the same breed again. The only exception was in owners of French Bulldogs, where the extent of experienced health problems decreased the intention to acquire the same breed again.

The two dog breeds with extreme physical features included in this study have enjoyed immense popularity during the last decade, which could be a sign of more general trends in preferences for some types of dogs: for example, a trend that celebrates the fashionability of the dog rather than its functional attributes [14].

In our study, we found indications to support the trend to put less emphasis on functional attributes, as owners of French Bulldogs and Cavalier King Charles Spaniels seemed to prioritize the dog’s distinctive appearance prior to acquisition, and owners who prioritized this type of feature showed higher levels of attachment to their dogs. This suggests that acquiring a dog based on its physical appearance, as owners of French Bulldogs and Cavalier King Charles Spaniels did in this study, does not necessarily detract from its value as an object of attachment–the two may even be linked in that the physical appearance of these dogs–e.g. infantile facial features such as large forehead, large and low-lying eyes, and bulging cheeks–may contribute to the attachment process by acting as releasers of parental nurturing and caregiving behavior [22].

Using a term from Beverland and others [24], owners of Chihuahuas, French Bulldogs and Cavalier King Charles Spaniels might represent examples of ‘extrinsically motivated’ owners. These are owners who acquire dogs as a means of obtaining status and attention from other people, due to the distinctiveness or cuteness of the dog (in the case of French Bulldogs or Cavalier King Charles Spaniels), or as part of fashion (in the case of Chihuahuas), and may often perceive their dog as helpless and in need of care and control. In contrast, ‘intrinsically motivated’ owners are more likely to appreciate the individuality and autonomy of the dog and, as Ahuvia [56] further notes, they may perceive their dogs as friends rather than children in need of fully controlled limits. Based on the results from this study, we suggest that owners of Cairn Terriers seemingly act on intrinsic motivations when acquiring a dog. They showed a higher propensity to answer “I/we have owned a dog of this breed before”, and “I/we got the dog from a breeder with several breeding dogs”, and they were more motivated by breed attributes, such as health, and less motivated by the distinctive appearance compared with owners of the other three dog breeds.

That humans can and do form strong attachments to animals is widely accepted in the literature on human-animal relations, although theoretical consistency in relation to the ideas, concepts, and definitions that underpin attachment to companion animals is lacking [25]. Previous studies have examined connections between different human and dog characteristics and levels of attachment between owners and their dogs but no coherent picture has emerged from this line of research. Dotson and Hyatt [57] found that owners of purebred dogs have stronger attachments to their dogs, while Marinelli and others [58] found the opposite.

The results from the current study suggest that one possible reason for these differing results is that purebred dogs are not a homogeneous category. Instead, different breeds may be acquired for different reasons and, hence, the level and type of attachment may be expected to differ even within groups of purebred dogs of similar size and function.

Based on their LAPS scores, this study supports the idea that people form strong emotional relationships with their dogs [25,59], but we also found that owners of all three dog breeds with either extreme physical features and/or a distinct cute looks showed higher levels of attachment to their dogs than owners of Cairn Terriers. Interestingly, higher levels of health and behavior problems did not appear to have a negative impact on the quality of the owner-dog relationship for owners of Chihuahuas and Cavalier King Charles Spaniels. On the contrary, high levels of health and behavior problems with these two dog breeds were positively correlated with high levels of owner-dog attachment measured using the LAPS, a finding that suggests that caregiving behavior may reinforce the formation of strong owner attachments. While caregiving has been proposed as an explanation for why people are emotionally attached to their dogs [60–62] it could also help explain why high levels of health and behavior problems in the dog can generate strong attachment ties between owners and their dogs.

Kurdek [59] examined the extent to which dogs serve as attachment figures and identified characteristics of persons with strong attachments to their dog. He found that high levels of caregiving were associated with high levels of owners’ attachment to their dogs and speculated that this association between caregiving and attachment might become self-reinforcing: that, ‘the activities involved in the array of caregiving might initially provide opportunities for owners to become attached to their pet dogs, that attachment itself may then later provide one motivation for sustaining those caregiving activities’ (p. 262). Further supporting this argument, Meyer and Forkman [63] found that the level of social fear found in the Dog Mentality Assessment was the only aspect of dog personality measured in the assessment that correlated with the emotional closeness felt by owners. One explanation for this correlation could be that owners of fearful dogs become more attached to their dogs due to the extra care and protection they are perceived to need. Another explanation is that owners who are very attached to their dogs may be more likely to respond to fearful behaviors in a way that reinforces them, thereby inadvertently encouraging their display.

It seems plausible to suggest that dog breeds with the kinds of extreme features observed in this study may elicit a more distinctive kind of attachment from their owners than more ‘normal’ and healthy dogs, partly because of the requirements for extra care. Due to its extremely tiny and fragile body, for example, the Chihuahua likely evokes high levels of caregiving behavior, which is also indicated in this study by the high proportion of owners of Chihuahuas (70%) who “would do almost anything to take care of my dog”. The relationship between owners and their Chihuahuas could be defined in terms of a ‘need-dependency’ [64] in which the dog is viewed and treated as a child. That people often view their relationship with their dog as similar to those with children or other family members [60,65–66] could further extend the boundaries of how far people are willing to go in terms of caregiving due to the emotionally strong bonds that normally exist in families. In addition to this, we found evidence to support the idea that attachment can be related to some specific physical (and probably behavioral/temperamental) features of dogs, which was also found in previous studies, e.g. Archer and Monton [22].

Among owners of Cavalier King Charles Spaniels and French Bulldogs in this study, the distinctive appearance of the dogs was shown to be one motivational factor relevant for dog acquisition that was also found to be correlated with the level of owner-dog attachment. We did not differentiate between specific physical features in detail in our survey but, since owners of French Bulldogs and Cavalier King Charles Spaniels showed significant preferences for the distinctive appearances of their dog compared to what was found among owners of the other two dog breeds, we have reason to implicate selection for neotenic or paedomorphic features in these breeds.

Previous studies have found that a dog’s behavior and personality may influence levels of owner-dog attachment [26–27]. Another study showed that the resemblance of personality traits between owners and their cats and dogs seemed to affect levels of owner attachment [67]. Based on owners’ responses to the 23 LAPS items, the present study found lower levels of owner-dog attachment among owners of Cairn Terriers compared with owners of the three dog breeds with extreme physical features and/or a high level of cuteness examined here. Cairn Terriers are considered by some authors to be relatively aloof compared to many other dog breeds [68]. This suggests that they may show fewer signs of attachment to their owners, which could be part of the explanation for the comparatively low scores we obtained for owner to dog attachments. In line with this, a study in Japan [69] detected significantly elevated levels of oxytocin in the urine of dog owners who received greater amounts of visual attention (gaze) from their dog in experimental trials. When questioned, these owners also professed stronger attachments for their more attentive dogs, suggesting that owner attachment may be affected by specific bonding signals given by their dogs. On the other hand, other studies have found no correlation between the attachment of the dog to the human and of the human to the dog [70].

Differences in levels of attachment across owners of different breeds may also, at least partly, be explained by differences in the people who choose to acquire particular dogs. As we saw, there are clear differences in motivations among people who are attracted to different breeds. To fully study this, it would be necessary to link our findings with various socio-demographic characteristics in more detail or even to do some kind of psychological profiling of potential owners of the different breeds. This is an important subject for further study but is beyond the scope of the current paper. In the context of the current paper it is important to underline that the results presented stand up, even when controlling for socio-demographic variables that are potential confounders.

It is a potential limitation of the present study that the owners reported their purchase motivation at different time points (depending on the age of the dog when they responded to the questionnaire). This may have affected their recollection and given rise to differential levels of recall bias. We opted to take this into accounted in our analyses of purchase motivation by controlling for the dog’s age. While this is not a perfect measure of recall bias, it nevertheless is a relevant proxy, which makes it credible that the identified differences between dog type owners are real.

It will be important in future studies to assess human perceptions of other dog breeds with different extreme features, such as excessive skin folding or giant size, and to extend analysis beyond Denmark, although the existence of a complete registry of all owned dogs made this country an ideal setting for our initial study. The current study has demonstrated that there is clear and statistically significant variation between breeds in the reasons for acquiring them and the level of attachment to them. Care should therefore be taken when extrapolating the findings to other breeds or breed-types or to other countries, cultures or times.

## Conclusion

Motivations for acquiring a dog differ broadly among owners of the four dog breeds included in this study and our results indicate that some motivational factors, e.g. the dog’s distinctive appearance, could in part explain differences in the quality of owner-dog relationships. Our results indicate that prospective owners of particularly Chihuahuas and French Bulldogs do not prioritize welfare-related breed attributes, such as health, when acquiring a dog. Further, owners of Cavalier King Charles Spaniels and Chihuahuas who experienced higher levels of health and behavior problems with their dogs were found to have a stronger degree of attachment to them. Also, experiences with health and behavior problems with the dogs do not seem to, in all cases, have a negative impact on the owners’ probability of acquiring a dog of the same breed again. An exception to this was found for owners of French Bulldogs, in which the extent of experienced health and behavior problems was associated with decreased intention to acquire the same breed again.

In all, this study prompts the conclusion that the apparent paradox of people who love their dogs continuing to acquire dogs from breeds with breed-related welfare problems may not be perceived as a paradox from the point of view of prospective owners of breeds such as Chihuahuas and French Bulldogs. Thus apparently available information about the problems in these two breeds has not served to prevent their growing popularity because fundamental emotional responses to the phenotypic attributes of these breeds are highly effective positive motivators. These findings illustrate the need to find better ways to motivate prospective owners to demand dogs that do not suffer from welfare problems related to extreme conformation and inbreeding.

## Supporting information

S1 File Brenda Bonnet 2013a. (PDF)

S2 File Brenda Bonnet 2013b. (PDF)

S3 File Brenda Bonnet 2013c. (PDF)

S4 File Brenda Bonnet 2013d. (PDF)

S5 File Brenda Bonnet 2013e. (PDF)

S6 File Brenda Bonnet 2013f. (PDF)

S7 File Brenda Bonnet 2013g. (PDF)

S8 File Brenda Bonnet 2013h. (PDF)

S1 Table (PDF)

S1 Dataset (XLSX)
