# Supplementary material for: Why do people buy dogs with potential welfare problems related to extreme conformation and inherited disease? A representative study of Danish owners of four small dog breeds
Source: PLoS One. 2017 Feb 24;12(2):e0172091. doi: 10.1371/journal.pone.0172091 (PMC5325474; doi:10.1371/journal.pone.0172091)
Supplement: S3 File — (PDF) [file pone.0172091.s003.pdf]

Agria Insurance Data

Updated Dog Breed Statistics : 2006-2011

LIFE INSURANCE EVENTS: MORTALITY

Cavalier King Charles Spaniel

Supplement to Agria Dog Breed Profiles (www.agria.se) and Updates (1995-2006)

The rates and measures are based on data from Agria Pet Insurance, Sweden. The primary goal of this ongoing work has been to provide information to Breed Clubs in Sweden on health issues in their Breed. The original Breed Profiles were provided (free-of-charge) to the Swedish Kennel Club and to each Breed Club (80 Breeds; 1995-2002). The Updates 1995-2006 have also been shared widely (over 100 breeds), within Sweden and Denmark.

Every effort has been made to calculate statistics using unbiased and scientifically valid techniques. The data are, however, affected by terms and conditions of the insurance products, the enrolment of dogs in insurance, the owner's decisions regarding seeking and receiving veterinary care for their dog, the way in which veterinarians diagnose and treat illness, amongst other factors. Obviously, all of these influences and factors may change over time.

To minimize possible misunderstanding, essentially all material is presented as a comparison between the Breed and All Breeds, combined. It is assumed that changes or influences affecting the data are likely to be similar across breeds. As this is the second set of Updates, and more are possible in the future, it is believed that this comparative approach is the safest, in terms of preventing mis-interpretation or over-interpretation of findings.

These Dog Breed Statistics 2006-2011 are presented in a format essentially similar to the previous Updates 1995-2006. See below for a further description of the calculation of rates. *Readers will want to know if rates of disease in their Breed are increasing or decreasing over time.* Unfortunately, due to the changing nature of the database and insurance policies, only comparisons between rates for the Breed and All Breeds can be made. The relative rates (risks) compared to All Breeds in the earlier Updates (1995-2006) can be compared to those in this version (2006-2011). Of course, differences between the Breed and All Breeds maybe due to changes in the Breed, All Breeds or both. However, marked changes in Breed risk are worth noting given that All Breeds reflects over *1.35 million dog-years-at-risk.*

Agria Djurförsäkring

Background Information and Hints on Interpretation

Rates are based on dog-years-at-risk (DYAR) which take into account the actual time each dog was insured during the period (2006-2011). A dog insured for an entire year contributes 1 DYAR, a dog insured for only 6 months contributes 0.5 DYAR. Overall rates are expressed as the number of dogs dead, with a life insurance claim, per 10,000 DYAR. However, actual values for the rates are not given, only the comparison to rates in All Breeds. There is no differentiation as to whether the dog died or was euthanized.

To interpret the horizontal bar charts (Charts 1, 2, 3, 5, 7) comparing the Breed to All Breeds: The further to the right that the bar extends, the more common the condition (the higher the rate). If the Breed bar is approximately the same length as the bar for All Breeds, that condition causes death in the breed at approximately the rate as in All Breeds. For conditions where the Breed bar is longer than the All Breeds bar, the Breed is at increased risk of dying from that condition compared to All Breeds. If the Breed bar is shorter, then the Breed dies due to that condition less than All Breeds. Charts 4 and 6 quantify the risk in the Breed compared to All Breeds (Relative Risk). Note: no assessment of 'statistical significance' has been done, however due to the large number of dogs, small differences would likely be statistically significant.

Readers must balance all the presented information, together what is already known about health issues in the breed, to arrive at a sensible interpretation.

NOTE: Mortality rates include events where, most commonly, a veterinarian assigned the cause of death and some cases (generally acute or accidental death) where the owner and a witness confirmed the death of the dog. The maximum age to which a dog could be life insured varied somewhat across breeds and years. Certain restrictions of the insurance policies affect the statistics.

Approximate numbers of Dog-Years at Risk for the Breed and All Breeds:

	**Dog-Years-at-Risk (DYAR, average, yearly)**
**Cavalier King Charles Spaniel**	2000<5000
**All Breeds**	>1.35 million

Chart 1

Overall Mortality Rates 2006-2011

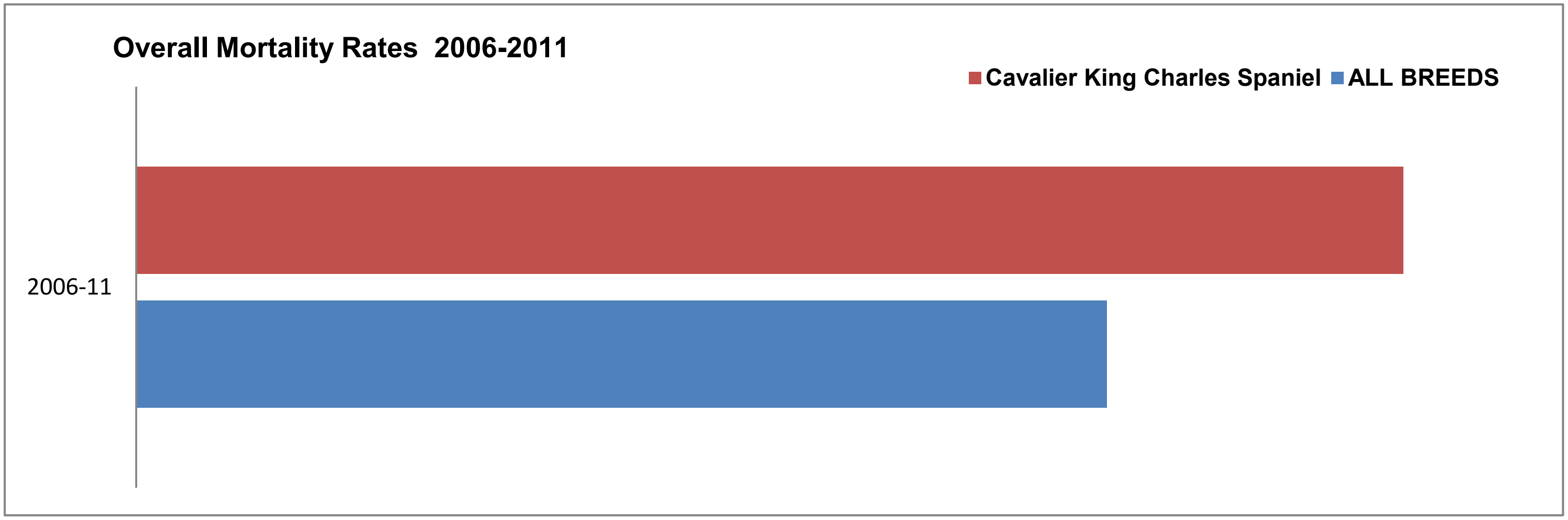

Relative risk of death :	Cavalier King Charles Spaniel	**2006-2011**
compared to	ALL BREEDS	1.3

Interpretation: *The mortality rate in Cavalier King Charles Spaniel was approximately 1.3 times higher than that for All Breeds.*

Interpretation:

Use the data on this page to get an overview of the health of the Breed compared to All Breeds, i.e. Is the mortality higher, lower or approximately the same as that for All Breeds?

Chart 2

Overall Mortality Rates for Males and Females: Cavalier King Charles Spaniel and All Breeds 2006-2011

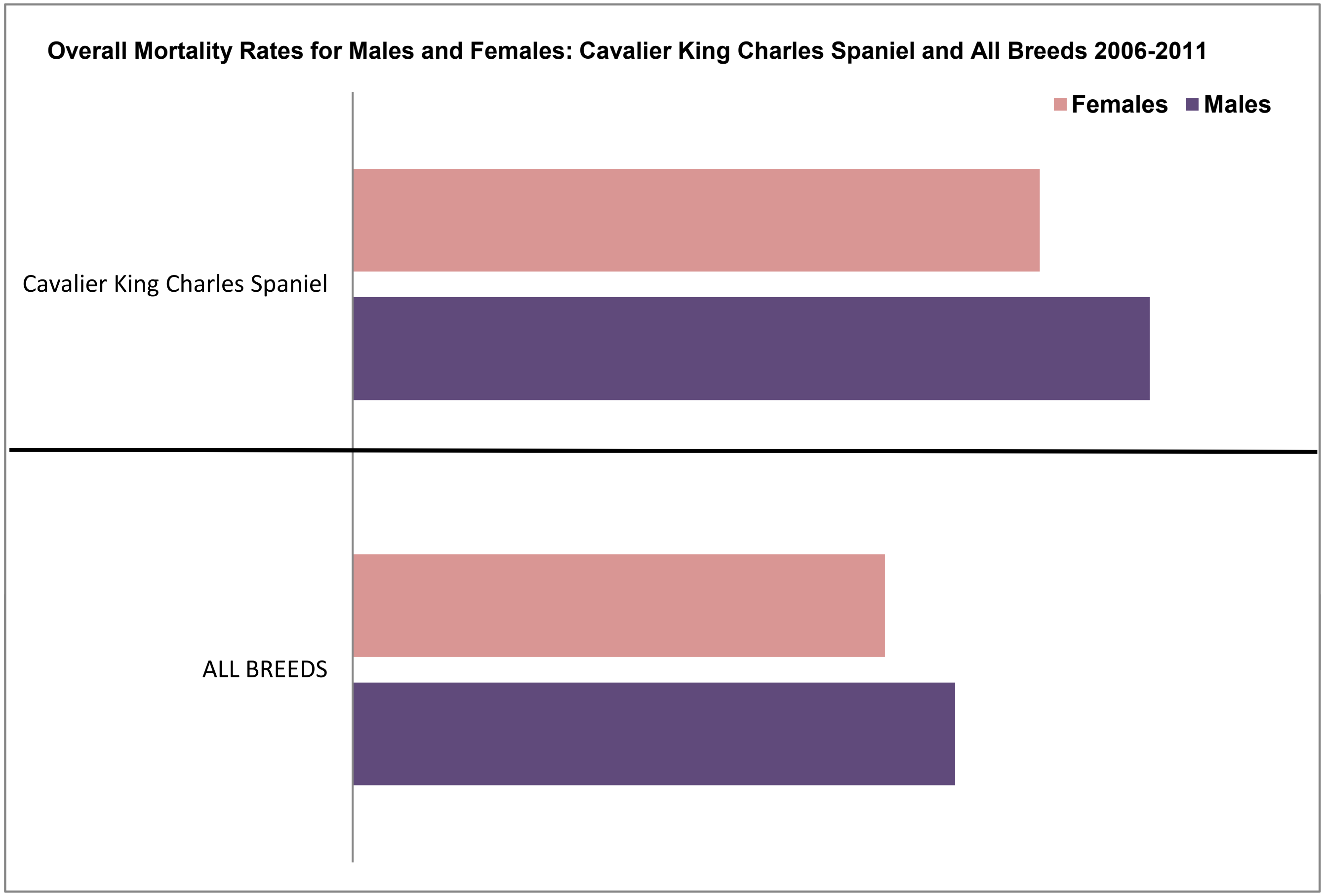

Interpretation:

Compare between sexes within Breed and for males and females of the Breed compared to All Breeds. If there are differences, consider general and specific causes of death (below) for explanations.

Median Age (years) at death*

(50% of dogs that died, died before this age, 50% after)

	Males	Females
**Cavalier King Charles Spaniel**	7.80	7.90
**ALL BREEDS**	6.60	7.00

Interpretation: *Do males and females of this Breed tend to die at a similar, earlier or later age (on average) compared to All Breeds?*

Chart 3

General Causes of Mortality: Rates in 2006-2011

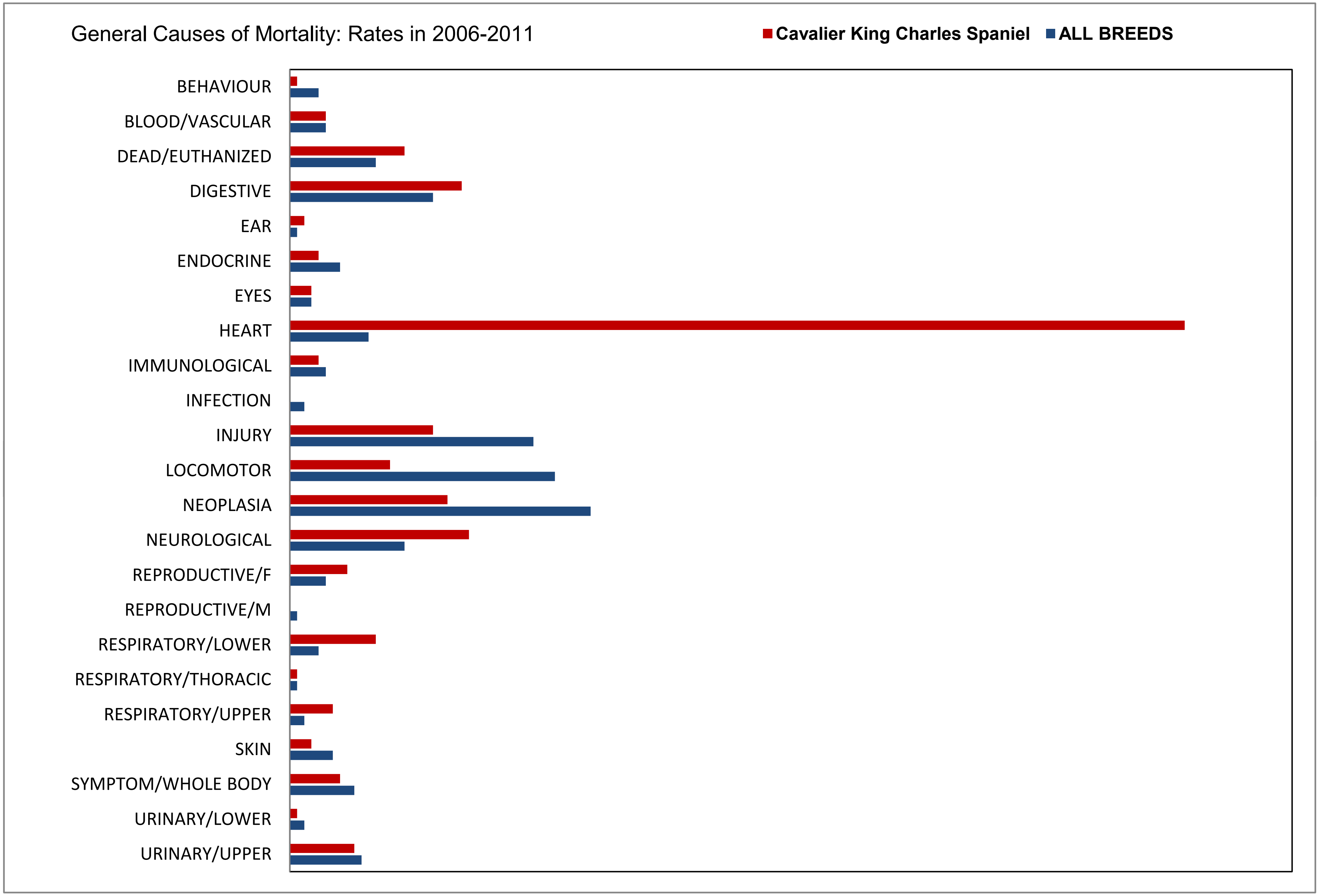

Chart 4

General Causes of Mortality Ordered by Relative Risk compared to All Breeds 2006-2011

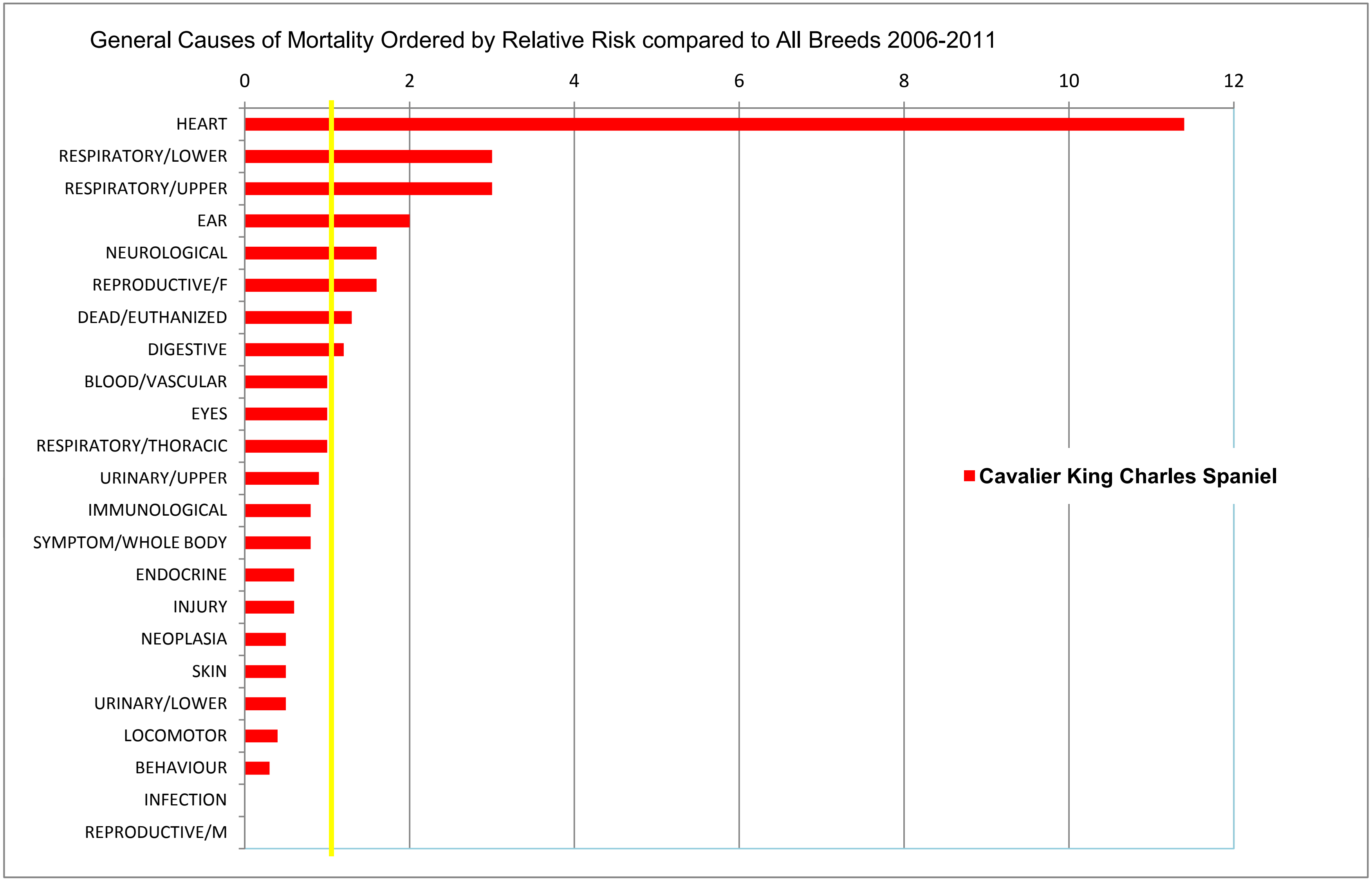

Interpretation:

The yellow line is the baseline risk for All Breeds; so, for those conditions where the red bar goes to the right of the yellow line, the breed is at increased risk compared to All Breeds. If the red bar goes to '2' it means that the risk in the Breed is approximately 2 times that for All Breeds.

Chart 5
Highest risk (most common) Specific Causes of Mortality (Diagnoses Level_1) 2006-2011

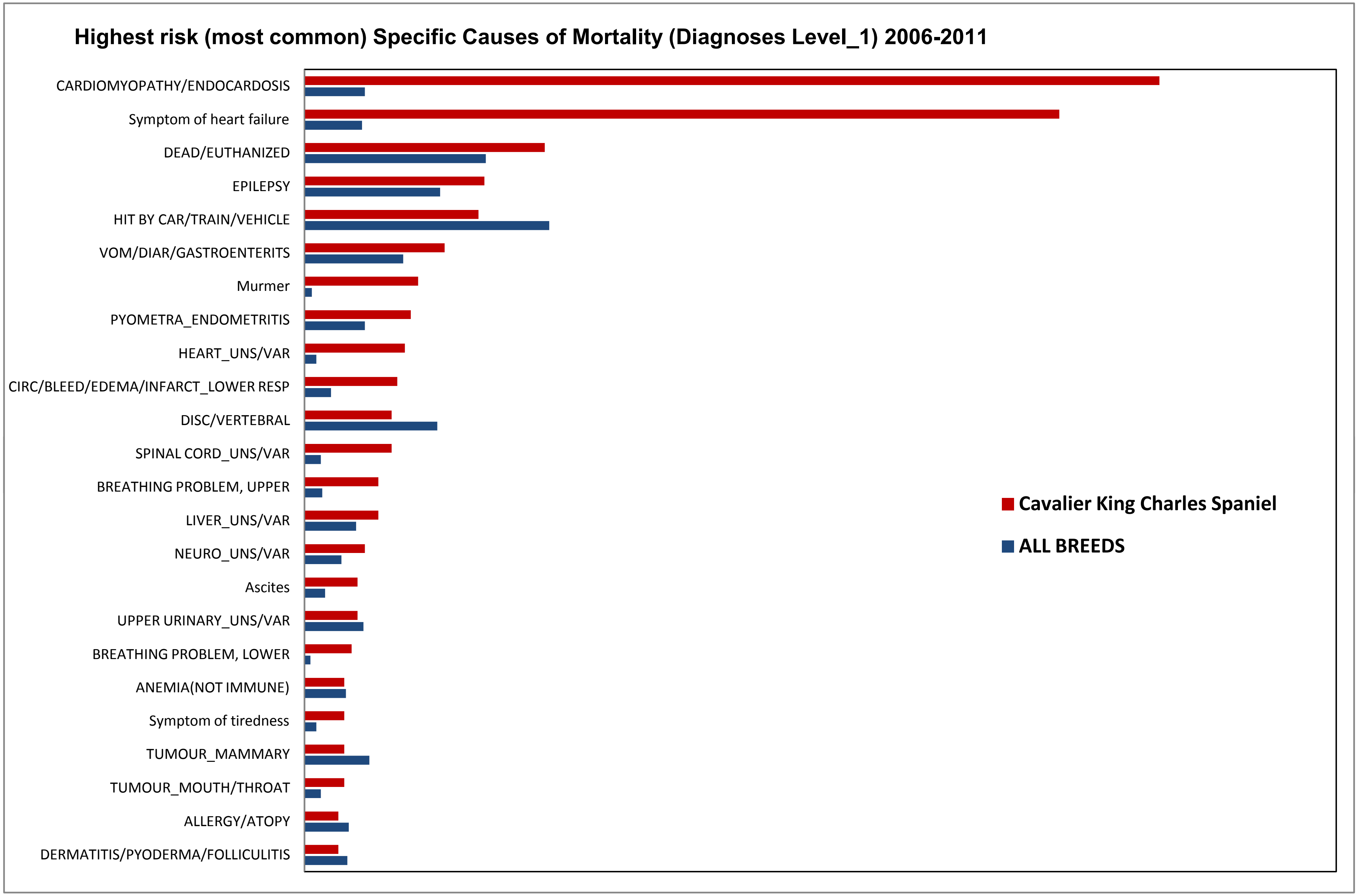

Chart 6

Specific Causes of Mortality Ordered by Relative Risk: Cavalier King Charles Spaniel compared to All Breeds 2006-2011

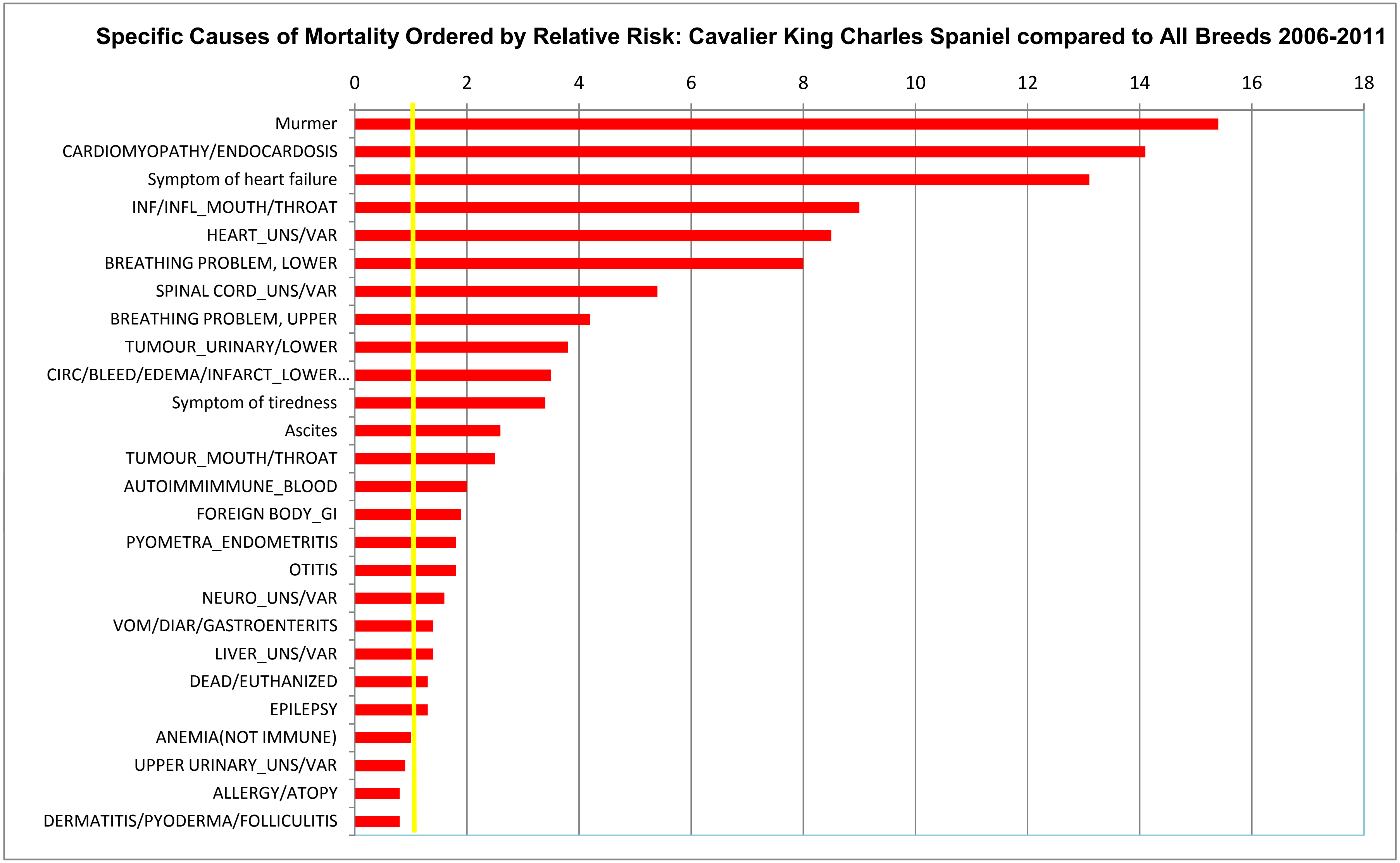

Interpretation: *NOTE: conditions with <4 deaths have been dropped. As for Chart 4. Note: Rare conditions that occur sporadically may appear as high relative risk (RR). Compare to previous chart(s) to help judge commonness of conditions with high RR in order to assess the importance of findings.*

Chart 7

Risk of Mortality for Locomotor Conditions 2006-2011

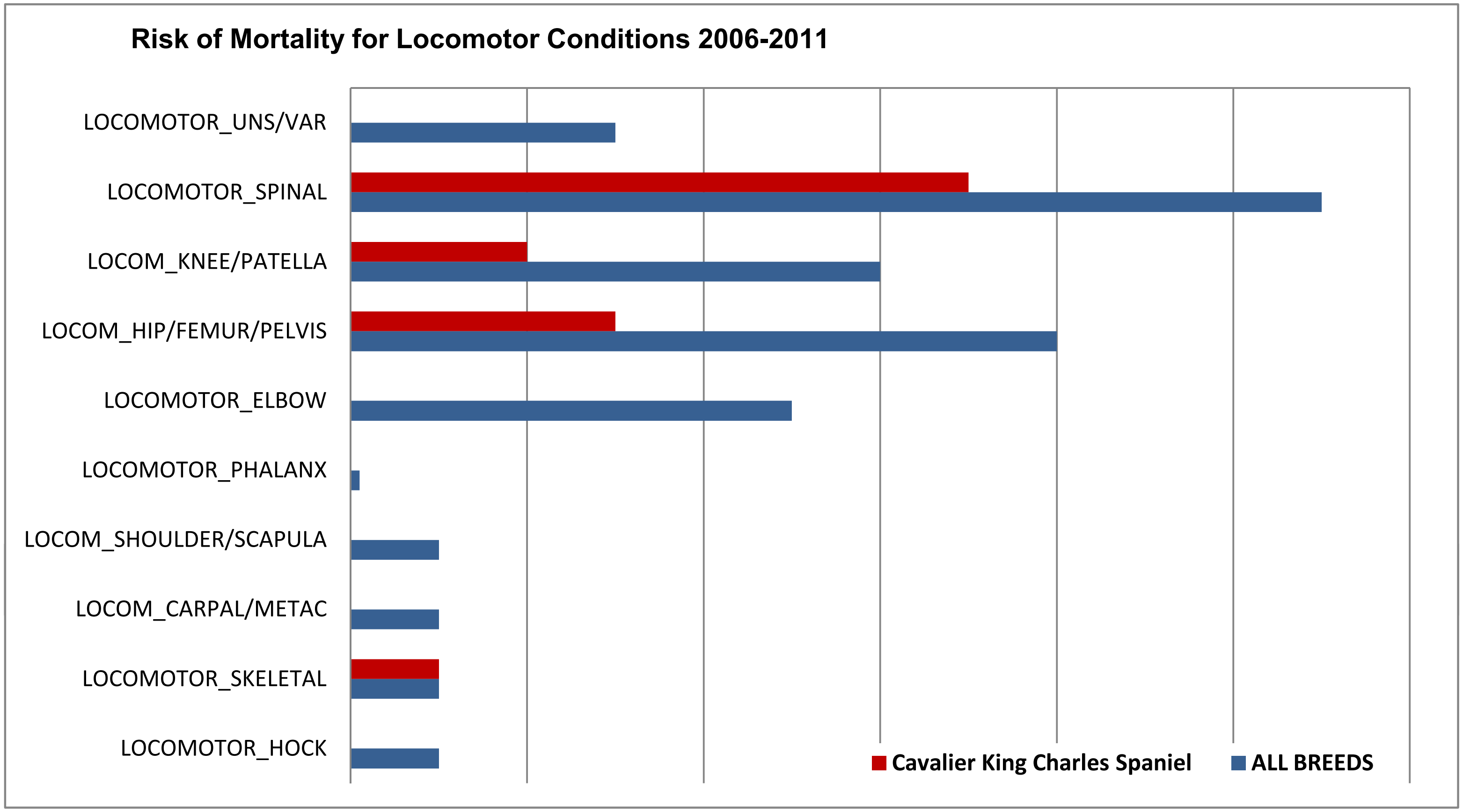

Note: These conditions are presented in order of decreasing commonness FOR VETERINARY CARE for All Breeds (to allow for direct comparison). 'UNS/VAR' means that the veterinarian did not specify a specific location or diagnosis or there were generalized locomotor problems.
