# Supplementary material for: Why do people buy dogs with potential welfare problems related to extreme conformation and inherited disease? A representative study of Danish owners of four small dog breeds
Source: PLoS One. 2017 Feb 24;12(2):e0172091. doi: 10.1371/journal.pone.0172091 (PMC5325474; doi:10.1371/journal.pone.0172091)
Supplement: S7 File — (PDF) [file pone.0172091.s007.pdf]

Agria Insurance Data

Updated Dog Breed Statistics : 2006-2011

VETERINARY CARE EVENTS

French Bulldog

Supplement to Agria Dog Breed Profiles (www.agria.se) and Updates (1995-2006)

The rates and measures are based on data from Agria Pet Insurance, Sweden. The primary goal of this ongoing work has been to provide information to Breed Clubs in Sweden on health issues in their Breed. The original Breed Profiles were provided (free-of-charge) to the Swedish Kennel Club and to each Breed Club (80 Breeds; 1995-2002). The Updates 1995-2006 have also been shared widely (over 100 breeds), within Sweden and Denmark.

Every effort has been made to calculate statistics using unbiased and scientifically valid techniques. The data are, however, affected by terms and conditions of the insurance products, the enrolment of dogs in insurance, the owner's decisions regarding seeking and receiving veterinary care for their dog, the way in which veterinarians diagnose and treat illness, amongst other factors. Obviously, all of these influences and factors may change over time.

To minimize possible misunderstanding, essentially all material is presented as a comparison between the Breed and All Breeds, combined. It is assumed that changes or influences affecting the data are likely to be similar across breeds. As this is the second set of Updates, and more are possible in the future, it is believed that this comparative approach is the safest, in terms of preventing mis-interpretation or over-interpretation of findings.

These Dog Breed Statistics 2006-2011 are presented in a format essentially similar to the previous Updates 1995-2006. See below for a further description of the calculation of rates; the key feature is that the material is relatively conservative in calculating the occurence of disease. That is, any dog is counted only once within any category. *Readers will want to know if rates of disease in their Breed are increasing or decreasing over time.* Unfortunately, due to the changing nature of the database and insurance policies, only comparisons between rates for the Breed and All Breeds can be made. The relative rates (risks) compared to All Breeds in the earlier Updates (1995-2006) can be compared to those in this version (2006-2011). Of course, differences between the Breed and All Breeds maybe due to changes in the Breed, All Breeds or both. However, marked changes in Breed risk are worth noting given that All Breeds reflects over *1.35 million dog-years-at-risk.*

Background Information and Hints on Interpretation

Rates are based on dog-years-at-risk (DYAR) which take into account the actual time each dog was insured during the period (2006-2011). A dog insured for an entire year contributes 1 DYAR, a dog insured for only 6 months contributes 0.5 DYAR. Overall rates are expressed as the number of dogs experiencing at least one VCE (see below) per 10,000 DYAR. However, actual values for the rates are not given, only the comparison to rates in All Breeds. For General Causes, a dog is recorded as having one or more events within a diagnostic category, so is counted only once within a category or diagnosis, but counted separately for different causes (diagnoses).

To interpret the horizontal bar charts (Charts 1, 2, 3, 6, 8) comparing the Breed to All Breeds: The further to the right that the bar extends, the more common the condition (the higher the rate). If the Breed bar is approximately the same length as the bar for All Breeds, the breed experiences the condition to the same extent as All Breeds. For conditions where the Breed bar is longer than the All Breeds bar, the Breed is at increased risk for that condition. If the Breed bar is shorter, then the Breed experiences that condition less than All Breeds. Charts 4 and 7 quantify the risk in the Breed compared to All Breeds (Relative Risk). Note: no assessment of 'statistical significance' has been done.

Readers must balance all the presented information, together what is already known about health issues in the breed, to arrive at a sensible interpretation.

NOTE: Veterinary Care Events (VCEs) are those visits to veterinarians for which the cost exceeded the deductible (self-risk) and a claim was processed by the insurance company. Dogs could be insured to any age, although the number of dogs insured declines at older ages. Certain restrictions of the insurance policies affect the statistics, e.g. behaviour problems are, in general, not reimbursable and are not included in these statistics.

Approximate numbers of Dog-Years at Risk for the Breed and All Breeds:

	Dog-Years-at-Risk (DYAR, average, yearly)	
**French Bulldog**	500<1000	NOTE: FEW DOGS - INTERPRET CAUTIOUSLY
**All Breeds**	>1.35 million	

Chart 1

Overall Rates of Veterinary Care 2006-2011

NOTE: FEW DOGS - INTERPRET CAUTIOUSLY

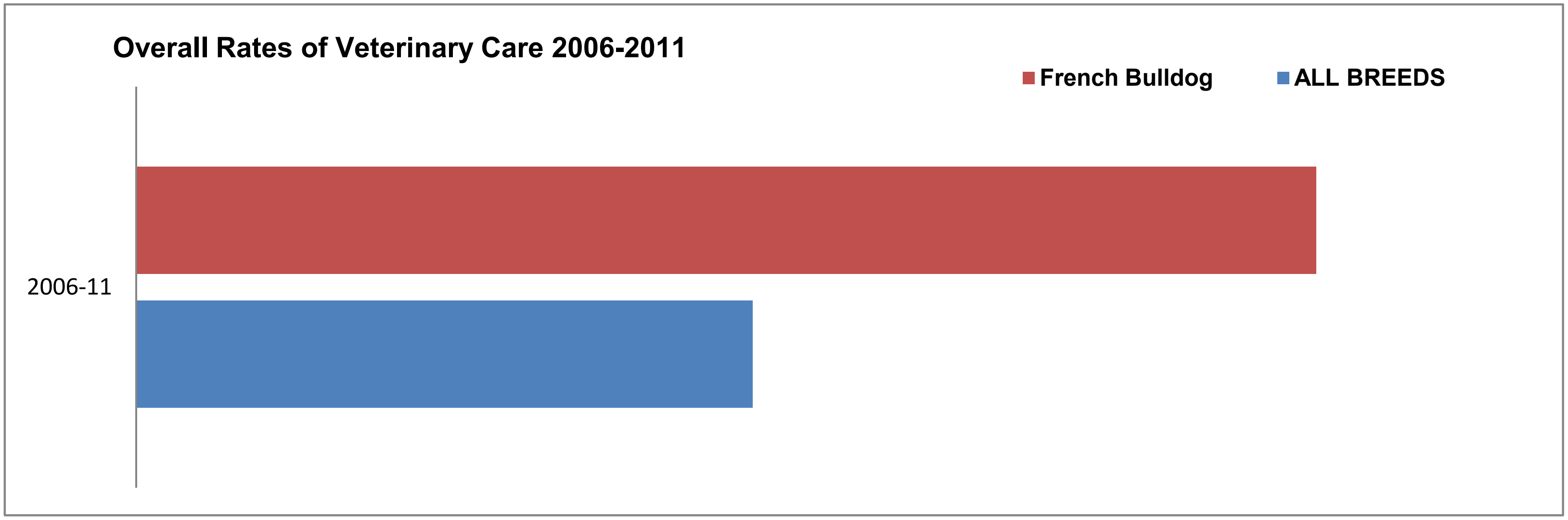

Relative risk of at least one VCE :	French Bulldog	**2006-2011**
compared to	ALL BREEDS	1.9

Interpretation:

The probability that a French Bulldog would have at least one VCE was 1.9 times that for All Breeds.

Percent of Insured Dogs with at least one VCE *

**French Bulldog**	38.7%
**ALL BREEDS**	31.8%

** Does not account for time at risk, interpret cautiously.*

Interpretation: *Use the data on this page to get an overview of the health of the Breed compared to All Breeds, i.e. Is the rate of dogs with at least one* major *veterinary care higher, lower or approximately the same as that for All Breeds?*

Chart 2

NOTE: FEW DOGS - INTERPRET CAUTIOUSLY

Overall rates of VCEs for Males and Females: French Bulldog and All Breeds 2006-2011

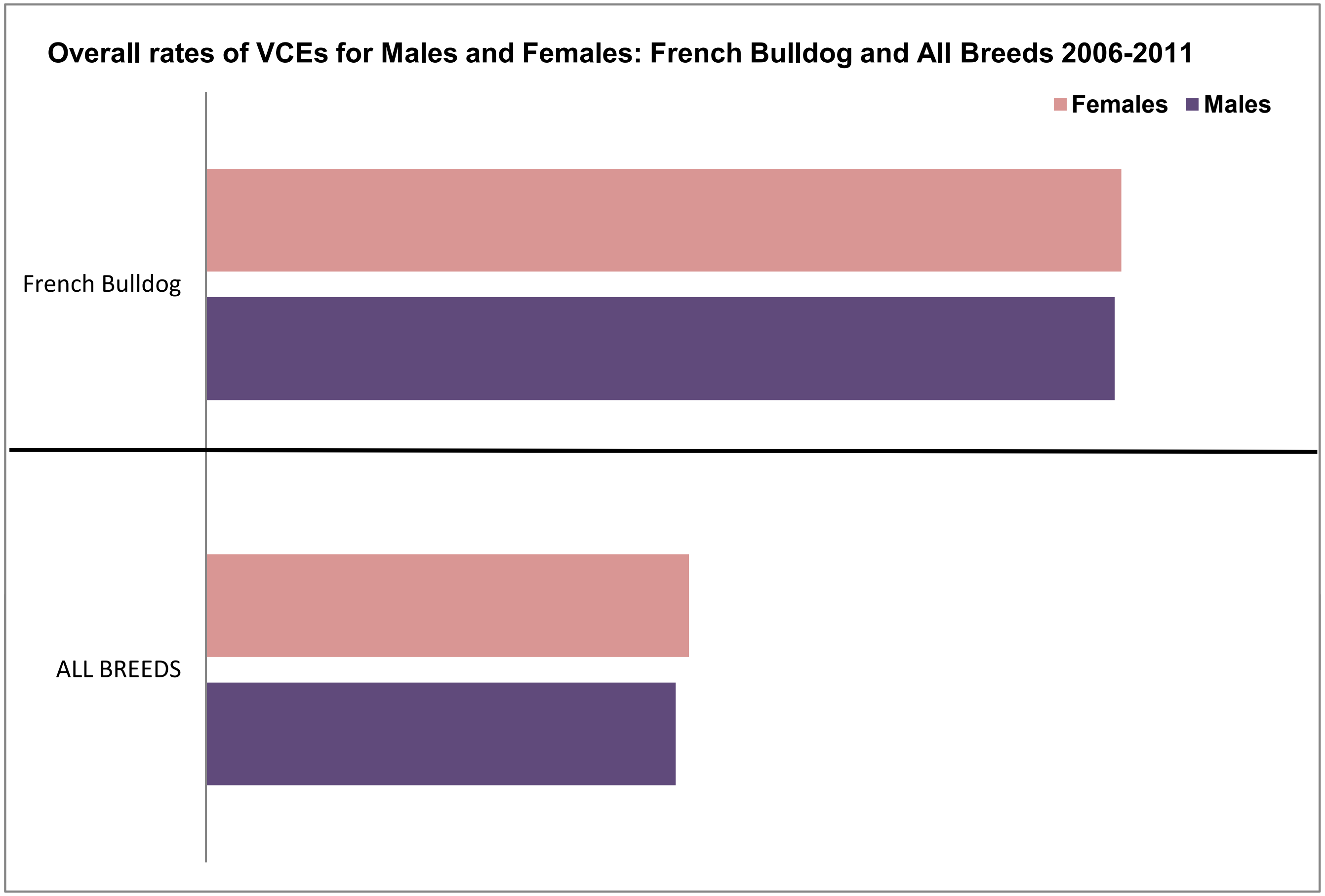

Interpretation:

Compare between sexes within Breed and for males and females of the Breed compared to All Breeds. If there are differences, consider general and specific causes of disease (below) for explanations.

Median Age (years) at 1st Veterinary Care Event*

(50% of dogs experience their first VCE before this age, 50% after.)

	Males	Females
**French Bulldog**	1.60	1.80
**ALL BREEDS**	4.90	5.60

** NOTE: In this database dogs may have been enrolled at different ages and, of course, can only experience a veterinary care event after they are enrolled.*

Interpretation: *Does is appear that dogs of this Breed tend to experience their first VCE at about the same age as All Breeds, or earlier or later?*

Chart 3

General Causes of VCEs : Rates in 2006-2011

NOTE: FEW DOGS - INTERPRET CAUTIOUSLY

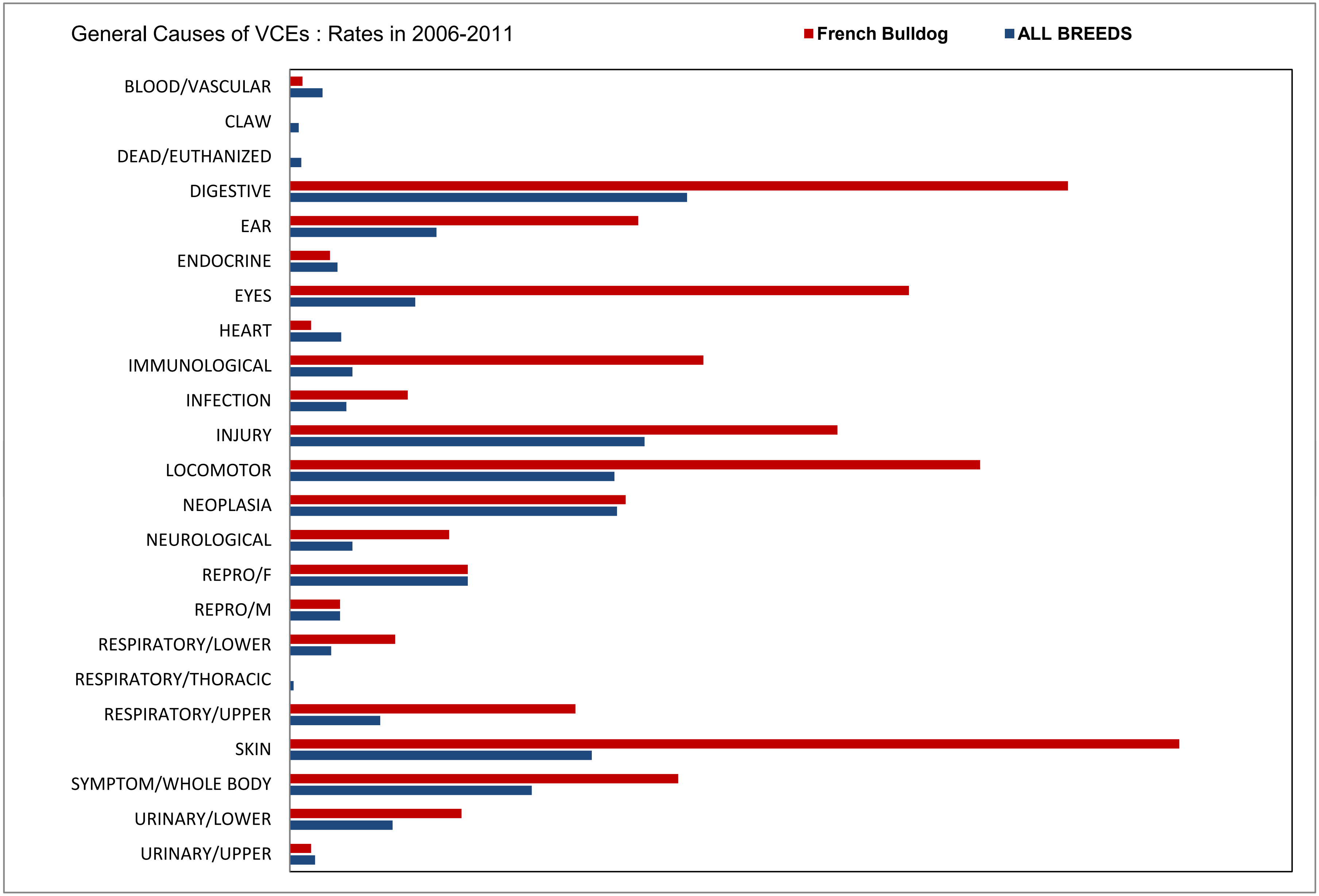

Chart 4

NOTE: FEW DOGS - INTERPRET CAUTIOUSLY

General Causes of VCEs Ordered by Relative Risk compared to All Breeds 2006-2011

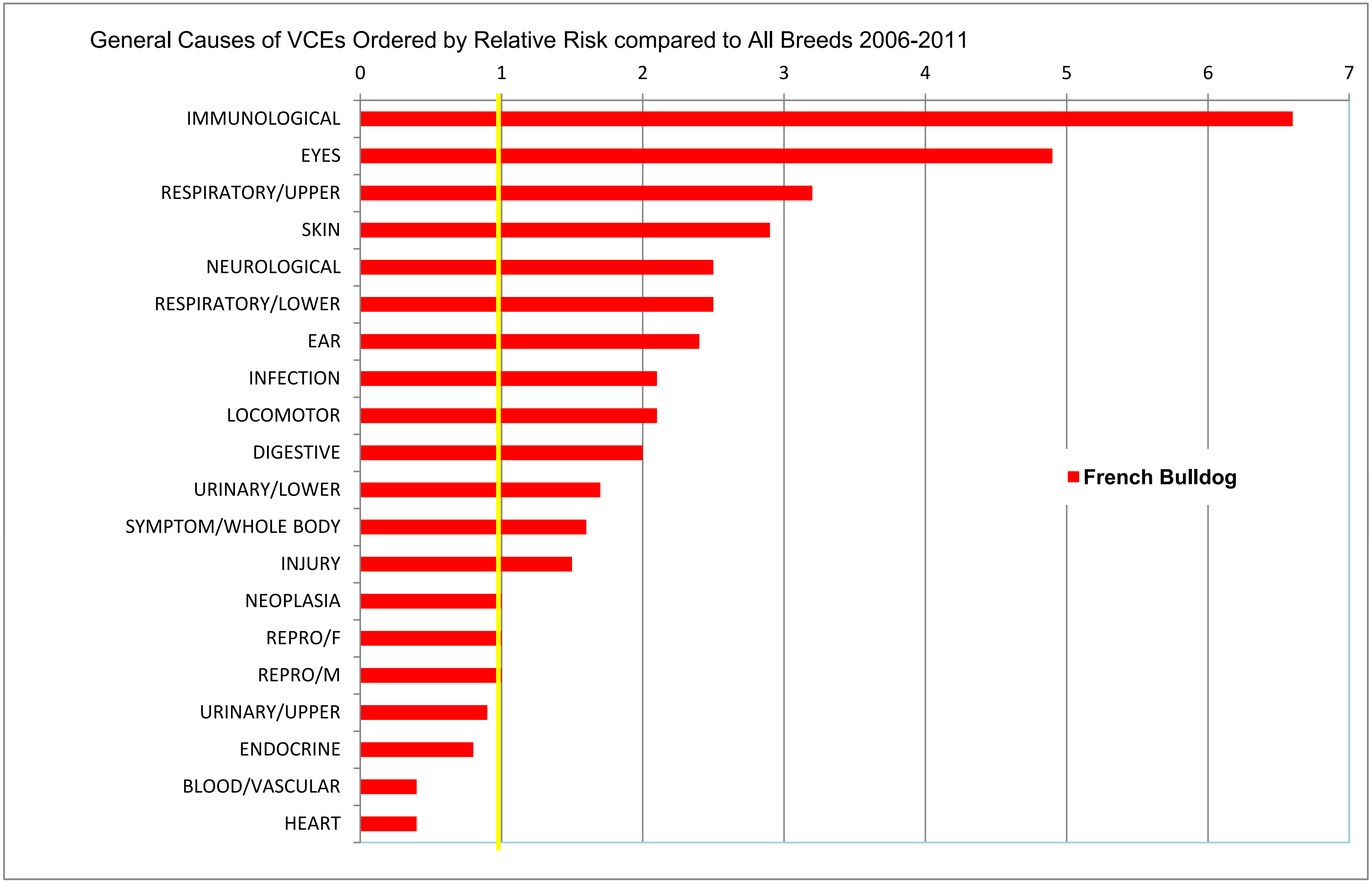

Interpretation:

The yellow line is the baseline risk for All Breeds; so, for those conditions where the red bar goes to the right of the yellow line, the breed is at increased risk compared to All Breeds. If the red bar goes to '2' it means that the risk in the Breed is approximately 2 times that for All Breeds.

Chart 5

NOTE: FEW DOGS - INTERPRET CAUTIOUSLY

Percent of Insured Dogs: General Causes of VCEs 2006-2011: French Bulldog

Interpretation: *Describes the proportion of all dogs that had at least one VCE within the specific system. Dogs are counted only once within a system, regardless of multiple claims, but may be counted in more than one system. NOTE: this does not account for varying times at risk across dogs and should, therefore, be used only as a very rough approximation of occurrence in insured dogs.*

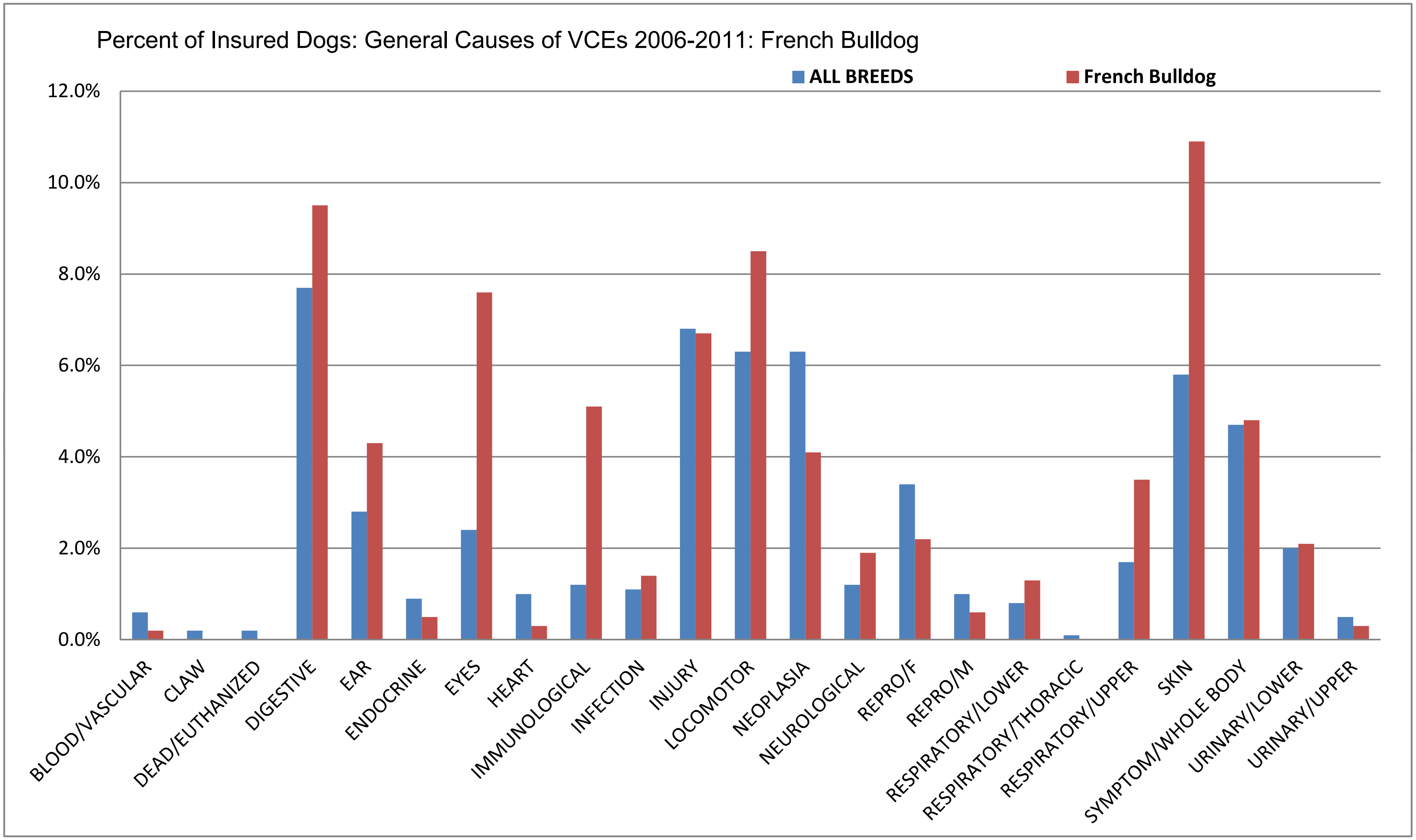

Chart 6

NOTE: FEW DOGS - INTERPRET CAUTIOUSLY

Highest risk (most common) Specific Causes of VCEs (Diagnoses Level_1) 2006-2011

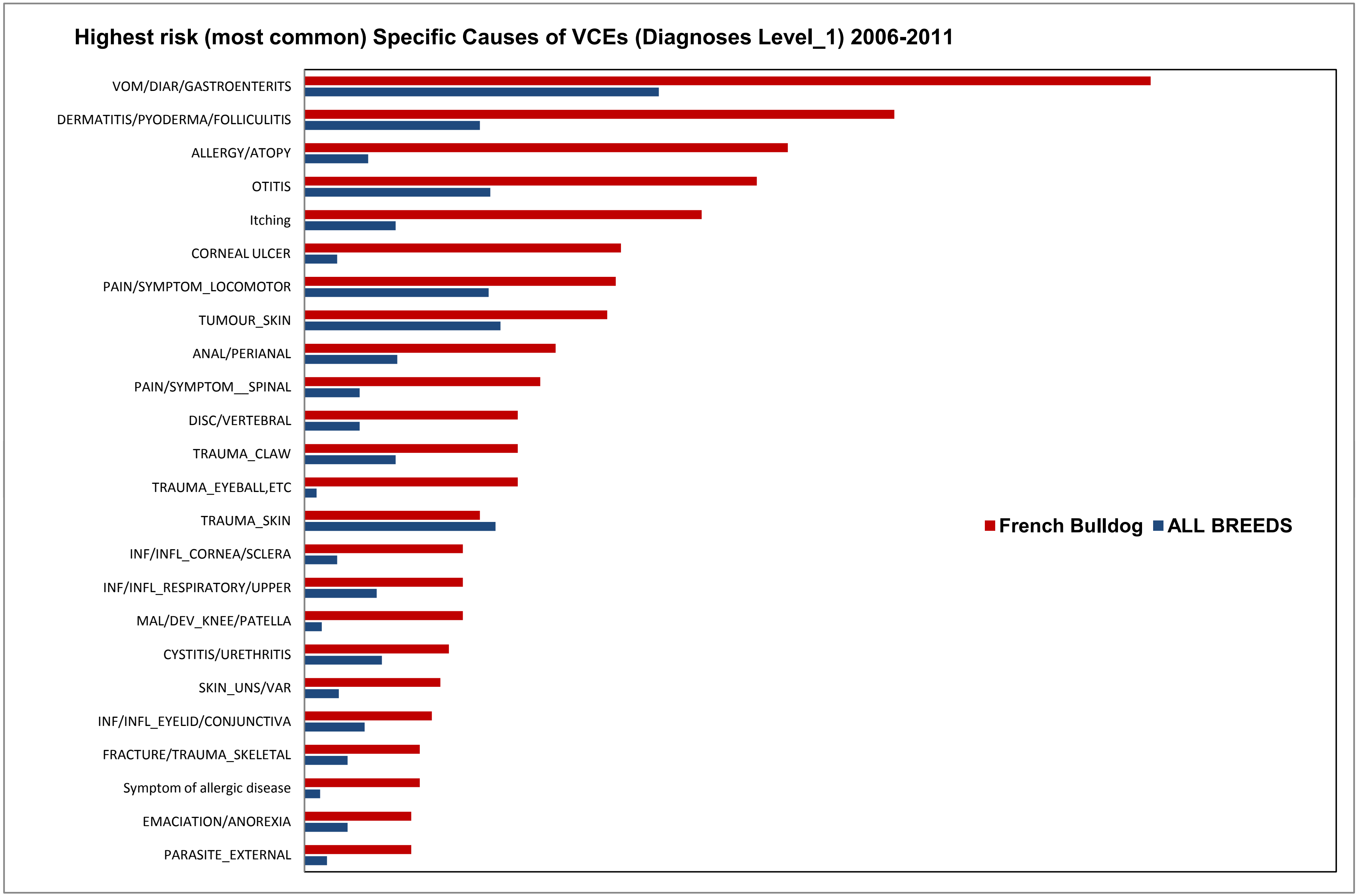

Chart 7

NOTE: FEW DOGS - INTERPRET CAUTIOUSLY

Specific Causes of VCEs Ordered by Relative Risk: French Bulldog compared to All Breeds 2006-2011

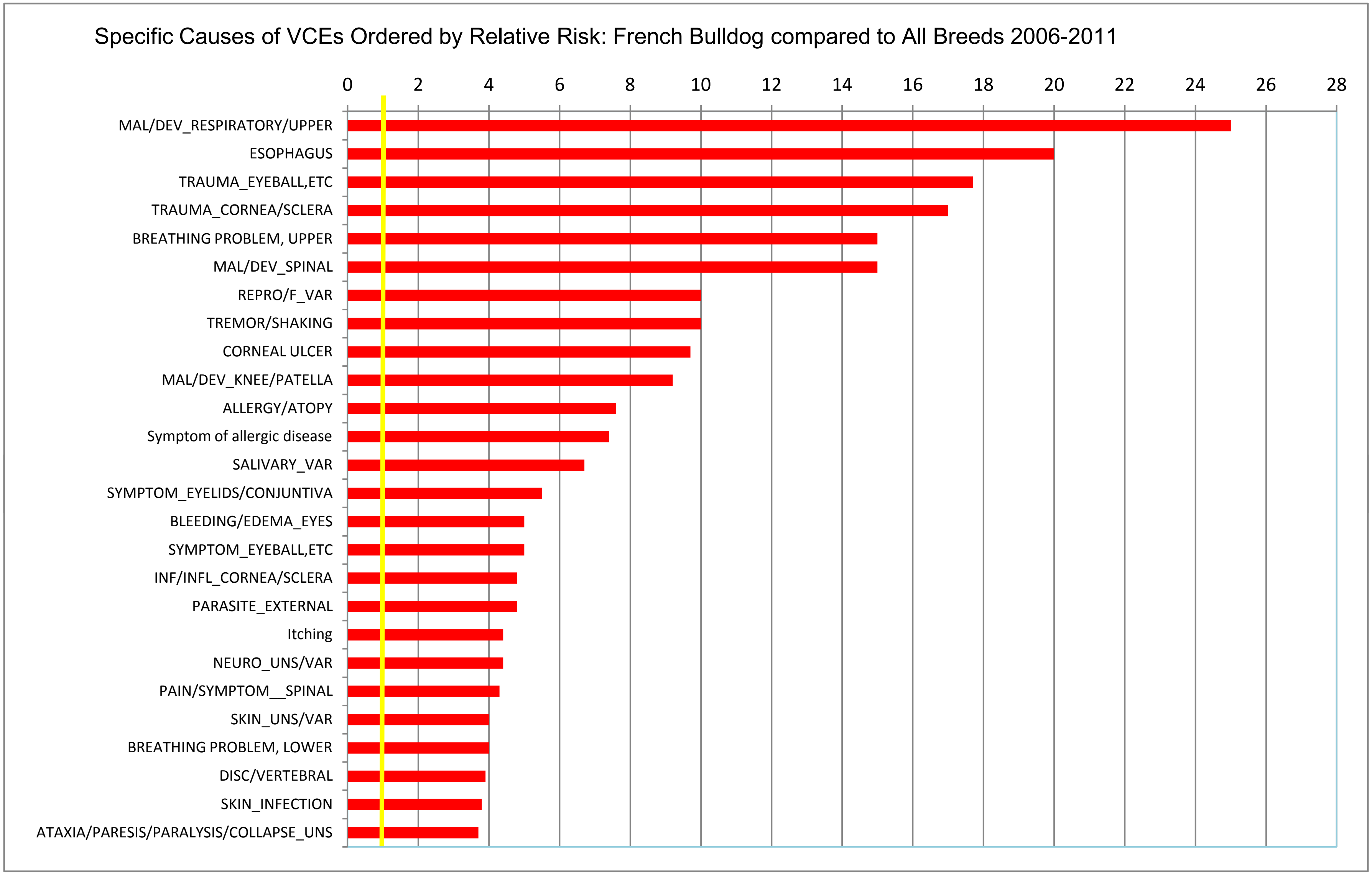

Interpretation: *As for Chart 4. Note: Rare conditions that occur sporadically may appear as high relative risk (RR). Compare to previous chart(s) to help judge commonness of conditions with high RR in order to assess the importance of findings.*

Chart 8

Risk of at least one VCE for Locomotor Conditions 2006-2011

NOTE: FEW DOGS - INTERPRET CAUTIOUSLY

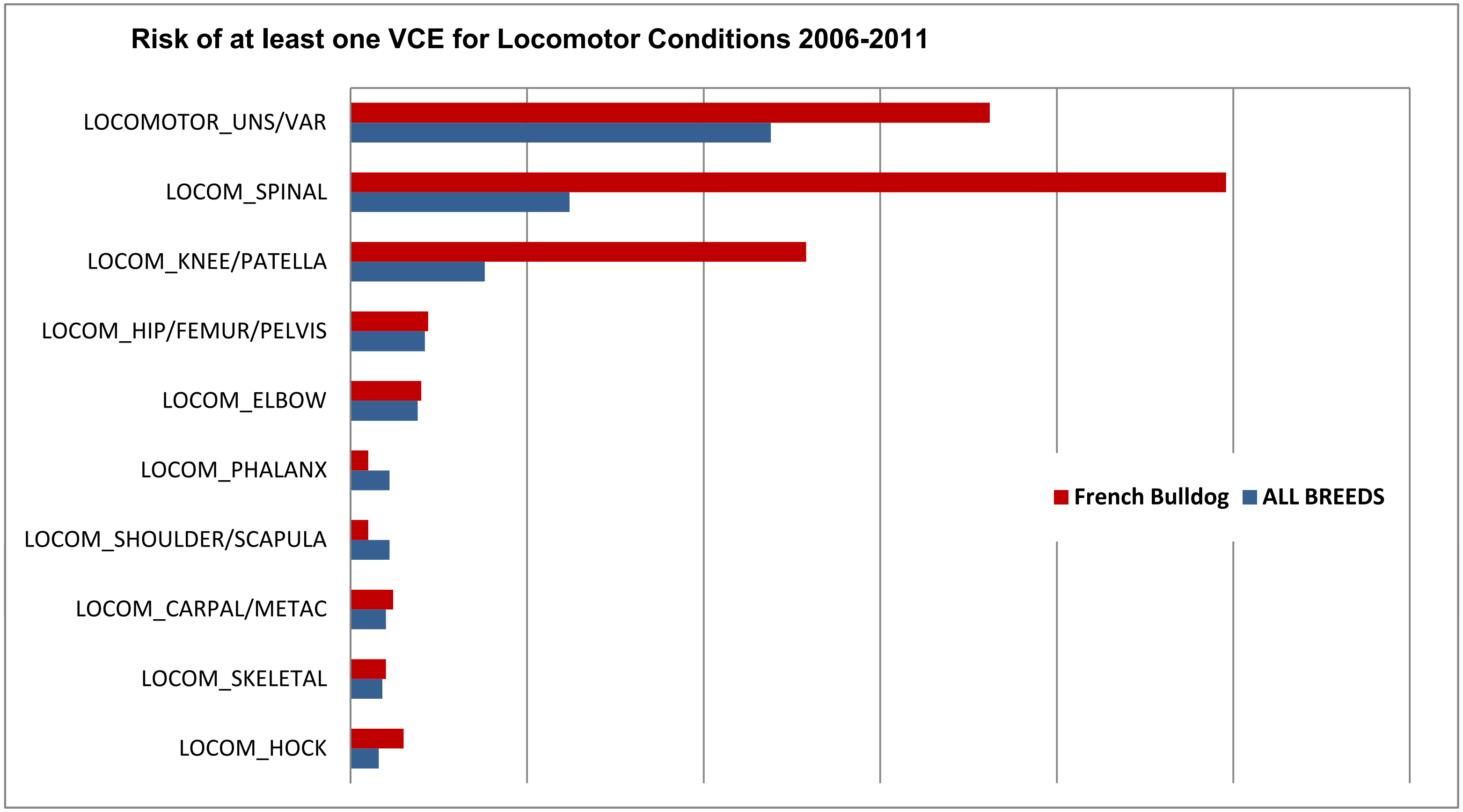

Note: These conditions are presented in order of decreasing commonness in All Breeds. 'UNS/VAR' means that the veterinarian did not specify a specific location or diagnosis or there were generalized locomotor problems.
