# Supplementary material for: Why do people buy dogs with potential welfare problems related to extreme conformation and inherited disease? A representative study of Danish owners of four small dog breeds
Source: PLoS One. 2017 Feb 24;12(2):e0172091. doi: 10.1371/journal.pone.0172091 (PMC5325474; doi:10.1371/journal.pone.0172091)
Supplement: S1 Table — (PDF) [file pone.0172091.s009.pdf]

Name	Question	Semantic description of codes	Composite variables/reverse scoring
Q1	What is your gender?	1=Female<br>2=Male<br>99999999=Missing	
Q2	What is your date of birth?	Year	
Q3	(Age categories)	1,00=12-29 years<br>2,00=30-39 years<br>3,00=40-49 years<br>4,00=50-59 years<br>5,00=60-69 years<br>7,00=70 years and more<br>99999999=Missing	
Q4	What is your highest level of education obtained?	1,00=Compulsory school<br>2,00=High school<br>3,00=Vocational<br>4,00=Higher education 2-4 years<br>5,00=Higher education, more than 4 years<br>99999999=Missing	
Q5	(Population density)	1,00=Densely populated area<br>2,00=Medium close (largest city over 40000)<br>3,00=Medium close (largest city under 40000)<br>4,00=Sparsely pop. (largest city over 15000)<br>5,00=Sparsely pop. (largest city under 15000)<br>99999999=Missing	
Q6	(Household composition)	1,00=Lives alone<br>2,00=Two adults<br>3,00=Family with child/children<br>99999999=Missing	

Q7	In what type of accommodation do you live?	1=In flat<br>2=In house with garden<br>3=In farm/house in the countryside	
Q8	What breed is/was your dog?	1=Cairn terrier<br>2=Cavalier King Charles Spaniel<br>3=Chihuahua<br>4=French bulldog<br>5=Mixed breed<br>6=I have not had any of these breeds within the last 5 years<br>99999999=Missing	
Q9	How old is your dog?	1,00=0-12 months<br>2,00=1 year<br>3,00=2 years<br>4,00=3 years<br>5,00=4 years<br>6,00=5 years<br>7,00=More than 5 years<br>99999999=Missing	
Q10	(The choice of breed was incidental)	0=Not chosen<br>1=Chosen<br>99999999=Missing	
Q11	How much planning was there before the purchase of the dog?	1=It had been planned for a year or more<br>2=It had been planned for some time<br>3=It went fast as I/we first started thinking about getting a dog<br>4=There was no real planning<br>5=Don't know/don't remember<br>99999999=Missing	
Q12	(What influenced your choice to purchase a dog? I/we read books about dogs)	0=Not chosen<br>1=Chosen<br>99999999=Missing	

Q13	(What influenced your choice to purchase a dog? I/we contacted professionals to learn more about the dog)	0=Not chosen<br>1=Chosen<br>99999999=Missing	
Q14	(What influenced your choice of dog breed? I/we read books about this breed)	0=Not chosen<br>1=Chosen<br>99999999=Missing	
Q15	(What influenced your choice of dog breed? I/we contacted professionals to learn more about different dog breeds)	0=Not chosen<br>1=Chosen<br>99999999=Missing	
Q16	(Professional advice)		Q12+Q13+Q14+Q15
Q17	(What influenced your choice of dog breed? I/we have had this breed before)	0=Not chosen<br>1=Chosen<br>99999999=Missing	
Q18	(What influenced your choice of dog breed? Friends/colleagues/family recommended this breed)	0=Not chosen<br>1=Chosen<br>99999999=Missing	
Q19	(Where did you get or buy your dog from? From a breeder with several breeding dogs)	0=Not chosen<br>1=Chosen<br>99999999=Missing	
Q20	(Where did you get or buy your dog from? From a breeder/family with only the bitch who is the mother of the dog)	0=Not chosen<br>1=Chosen<br>99999999=Missing	
Q21	(Where did you get or buy your dog from? I/we got the dog from a previous owner)	0=Not chosen<br>1=Chosen<br>99999999=Missing	
Q22	(Where did you get or buy your dog from? Other)	0,00=Not chosen<br>1,00=Chosen	

Q23	To what degree did the dog's facial expression influence the choice of dog?	1=Not at all<br>2=Low degree<br>3=To some degree<br>4=High degree<br>5=Very high degree<br>99999999=Missing	
Q24	To what degree did the dog's overall appearance affect the choice of your dog?	1=Not at all<br>2=Low degree<br>3=To some degree<br>4=High degree<br>5=Very high degree<br>99999999=Missing	
Q25	To what degree did the fact that the dog was different/unique affect the choice of your dog?	1=Not at all<br>2=Low degree<br>3=To some degree<br>4=High degree<br>5=Very high degree<br>99999999=Missing	
Q26	To what degree did the color of the dog affect the choice of your dog?	1=Not at all<br>2=Low degree<br>3=To some degree<br>4=High degree<br>5=Very high degree<br>99999999=Missing	
Q27	To what degree did the dog's breed affect the choice of your dog?	1=Not at all<br>2=Low degree<br>3=To some degree<br>4=High degree<br>5=Very high degree<br>99999999=Missing	

Q28	To what degree did the characteristics/behavior of the breed affect the choice of your dog?	1=Not at all<br>2=Low degree<br>3=To some degree<br>4=High degree<br>5=Very high degree<br>99999999=Missing	
Q29	To what degree did the health of the breed affect the choice of your dog?	1=Not at all<br>2=Low degree<br>3=To some degree<br>4=High degree<br>5=Very high degree<br>99999999=Missing	
Q30	To what degree did the fact that it was easy to find a dog of this breed affect the choice of your dog?	1=Not at all<br>2=Low degree<br>3=To some degree<br>4=High degree<br>5=Very high degree<br>99999999=Missing	
Q31	To what degree did the fact that the dog was a bargain (it was a fair price) affect the choice of your dog?	1=Not at all<br>2=Low degree<br>3=To some degree<br>4=High degree<br>5=Very high degree<br>99999999=Missing	
Q32	(Distinctive appearance)		Q23+Q24+Q25+Q26
Q33	(Breed attributes)		Q27+Q28+Q29
Q34	(Convenience)		Q30+Q31

Q35	To what degree did the dogs' personality affect the choice of your dog?	1=Not at all<br>2=Low degree<br>3=To some degree<br>4=High degree<br>5=Very high degree<br>99999999=Missing	
Q36	Has your dog experienced any problems with vomiting and/or diarrhea, where it did not require a vet visit?	1=Never<br>2=One or a few times<br>3=Many times<br>4=Don't know<br>99999999=Missing	
Q37	Has your dog experienced any skin changes, ear problems, itching, problems with the anal glands, where it did not require a vet visit?	1=Never<br>2=One or a few times<br>3=Many times<br>4=Don't know<br>99999999=Missing	
Q38	Has your dog experienced any problems with coughing, wheezing or strange breathing sounds, where it did not require a vet visit?	1=Never<br>2=One or a few times<br>3=Many times<br>4=Don't know<br>99999999=Missing	
Q39	Has your dog experienced any cramps, unsteadiness, problems with balance, where it did not require a vet visit?	1=Never<br>2=One or a few times<br>3=Many times<br>4=Don't know<br>99999999=Missing	
Q40	Has your dog experienced any behavioral problems such as aggression, uncleanliness, fear of noises or of being alone, where it did not require a vet visit?	1=Never<br>2=One or a few times<br>3=Many times<br>4=Don't know<br>99999999=Missing	

Q41	Has your dog been to the vet because of gastrointestinal problems (e.g. vomiting or diarrhea)?	1=Never<br>2=One or a few times<br>3=Many times<br>4=Don't know<br>99999999=Missing	
Q42	Has your dog been to the vet because of skin problems (e.g. skin changes, itching, otitis, problems with the anal glands)?	1=Never<br>2=One or a few times<br>3=Many times<br>4=Don't know<br>99999999=Missing	
Q43	Has your dog been to the vet because of respiratory problems (e.g. coughing, wheezing or strange breathing sounds)?	1=Never<br>2=One or a few times<br>3=Many times<br>4=Don't know<br>99999999=Missing	
Q44	Has your dog been to the vet because of disease of the brain or in other parts of the nervous system (e.g. epilepsy, slipped disc)?	1=Never<br>2=One or a few times<br>3=Many times<br>4=Don't know<br>99999999=Missing	
Q45	Has your dog been to the vet because of problems with the heart?	1=Never<br>2=One or a few times<br>3=Many times<br>4=Don't know<br>99999999=Missing	
Q46	Has your dog been to the vet because of behavioral problems such as aggression, uncleanliness, fear of noises or of being alone?	1=Never<br>2=One or a few times<br>3=Many times<br>4=Don't know<br>99999999=Missing	
Q47	(Number of frequently occurring problems not requiring veterinary assistance)		Q36+Q37+Q38+Q39 +Q40

Q48	(Number of problems requiring veterinary assistance many times)		Q41+Q42+Q43+Q44 +Q45+Q46
Q49	(Number of problems requiring/not requiring veterinary assistance)		Q47+Q48
Q50	How much money do you usually spend on annual veterinary bills?	1,00=0-999 DKR (0-150 USD)<br>2,00=1000-4999 DKR (151-759 USD)<br>3,00=5000 DKR or more (760 USD or more)<br>99999999=Missing	
Q51	Do you agree with the following statement: my dog means more to me than any of my friends?	1=Disagree<br>2=Partly disagree<br>3=Neither agree nor disagree<br>4=Partly agree<br>5=Agree<br>99999999=Missing	
Q52	Do you agree with the following statement: I often confide in my dog?	1=Disagree<br>2=Partly disagree<br>3=Neither agree nor disagree<br>4=Partly agree<br>5=Agree<br>99999999=Missing	
Q53	Do you agree with the following statement: dogs should, in my view, have the same rights and privileges as family members?	1=Disagree<br>2=Partly disagree<br>3=Neither agree nor disagree<br>4=Partly agree<br>5=Agree<br>99999999=Missing	
Q54	Do you agree with the following statement: my dog is my best friend?	1=Disagree<br>2=Partly disagree<br>3=Neither agree nor disagree<br>4=Partly agree<br>5=Agree<br>99999999=Missing	

Q55	Do you agree with the following statement: my feelings toward other people are quite often influenced by the way they respond to my dog?	1=Disagree<br>2=Partly disagree<br>3=Neither agree nor disagree<br>4=Partly agree<br>5=Agree<br>99999999=Missing	
Q56	Do you agree with the following statement: I love my dog because he/she is more loyal to me than most people in my life?	1=Disagree<br>2=Partly disagree<br>3=Neither agree nor disagree<br>4=Partly agree<br>5=Agree<br>99999999=Missing	
Q57	Do you agree with the following statement: I like to show other people pictures of my dog?	1=Disagree<br>2=Partly disagree<br>3=Neither agree nor disagree<br>4=Partly agree<br>5=Agree<br>99999999=Missing	
Q58	Do you agree with the following statement: to me my dog is just a dog?	1=Disagree<br>2=Partly disagree<br>3=Neither agree nor disagree<br>4=Partly agree<br>5=Agree<br>99999999=Missing	
Q59	Do you agree with the following statement: I love my dog, because it never judges me?	1=Disagree<br>2=Partly disagree<br>3=Neither agree nor disagree<br>4=Partly agree<br>5=Agree<br>99999999=Missing	

Q60	Do you agree with the following statement: my dog knows when I’m feeling bad?	1=Disagree<br>2=Partly disagree<br>3=Neither agree nor disagree<br>4=Partly agree<br>5=Agree<br>99999999=Missing	
Q61	Do you agree with the following statement: I often talk to other people about my dog?	1=Disagree<br>2=Partly disagree<br>3=Neither agree nor disagree<br>4=Partly agree<br>5=Agree<br>99999999=Missing	
Q62	Do you agree with the following statement: my dog understands me?	1=Disagree<br>2=Partly disagree<br>3=Neither agree nor disagree<br>4=Partly agree<br>5=Agree<br>99999999=Missing	
Q63	Do you agree with the following statement: I believe that the love for my dog helps me keep myself fit and healthy?	1=Disagree<br>2=Partly disagree<br>3=Neither agree nor disagree<br>4=Partly agree<br>5=Agree<br>99999999=Missing	
Q64	Do you agree with the following statement: dogs deserve as much respect as humans do?	1=Disagree<br>2=Partly disagree<br>3=Neither agree nor disagree<br>4=Partly agree<br>5=Agree<br>99999999=Missing	

Q65	Do you agree with the following statement: my dog and I have a very close relationship?	1=Disagree<br>2=Partly disagree<br>3=Neither agree nor disagree<br>4=Partly agree<br>5=Agree<br>99999999=Missing	
Q66	Do you agree with the following statement: I would do almost anything to take care of my dog?	1=Disagree<br>2=Partly disagree<br>3=Neither agree nor disagree<br>4=Partly agree<br>5=Agree<br>99999999=Missing	
Q67	Do you agree with the following statement: I quite often play with my dog?	1=Disagree<br>2=Partly disagree<br>3=Neither agree nor disagree<br>4=Partly agree<br>5=Agree<br>99999999=Missing	
Q68	Do you agree with the following statement: I consider my dog a good companion?	1=Disagree<br>2=Partly disagree<br>3=Neither agree nor disagree<br>4=Partly agree<br>5=Agree<br>99999999=Missing	
Q69	Do you agree with the following statement: my dog makes me happy?	1=Disagree<br>2=Partly disagree<br>3=Neither agree nor disagree<br>4=Partly agree<br>5=Agree<br>99999999=Missing	

Q70	Do you agree with the following statement: my dog is a part of my family?	1=Disagree<br>2=Partly disagree<br>3=Neither agree nor disagree<br>4=Partly agree<br>5=Agree<br>99999999=Missing	
Q71	Do you agree with the following statement: I am not particularly attached to my dog?	1=Disagree<br>2=Partly disagree<br>3=Neither agree nor disagree<br>4=Partly agree<br>5=Agree<br>99999999=Missing	
Q72	Do you agree with the following statement: having a dog helps to make me happy?	1=Disagree<br>2=Partly disagree<br>3=Neither agree nor disagree<br>4=Partly agree<br>5=Agree<br>99999999=Missing	
Q73	Do you agree with the following statement: I consider my dog a friend?	1=Disagree<br>2=Partly disagree<br>3=Neither agree nor disagree<br>4=Partly agree<br>5=Agree<br>99999999=Missing	
Q74	(Reverse score of Q58)		6-Q58
Q75	(Reverse score of Q71)		6-Q71
Q76	(LAPS Attachment scale)		Compute Q76 = Q51+Q52+Q53+Q54 +Q55+Q56+Q57+Q59+Q60+Q61 +Q62+Q63+Q64+Q65+Q66+Q67+Q68+Q

			69+Q70+Q72+Q73+<br>Q74+Q75
Q77	(Others in the household have main responsibility for the dog)	0,00=No<br>1,00=Yes	
Q78	(Others in the household have most contact with the dog)	0,00=No<br>1,00=Yes	
Q79	(Never travel on vacation)	0,00=No<br>1,00=Yes	
Q80	Do you brings the dog along on social visits?	1=No, never<br>2=Yes, sometimes<br>3=Yes, always<br>99999999=Missing	
Q81	(Plan to acquire a new dog after the current dog)	1,00=Same breed for sure<br>2,00=Maybe same breed<br>3,00=Not same breed<br>4,00=Not another dog<br>5,00=Get New dog: don't know	
Q82	(Plan to acquire the same breed after the current dog)	0,00=No, not for sure<br>1,00=Yes, for sure	
